# Mask wearing in community settings reduces SARS-CoV-2 transmission

**DOI:** 10.1073/pnas.2119266119

**Published:** 2022-05-31

**Authors:** Gavin Leech, Charlie Rogers-Smith, Joshua Teperowski Monrad, Jonas B. Sandbrink, Benedict Snodin, Robert Zinkov, Benjamin Rader, John S. Brownstein, Yarin Gal, Samir Bhatt, Mrinank Sharma, Sören Mindermann, Jan M. Brauner, Laurence Aitchison

**Affiliations:** ^a^Department of Computer Science, University of Bristol, Bristol BS8 1TH, United Kingdom;; ^b^External collaborator to Oxford Applied and Theoretical Machine Learning Group, University of Oxford, Oxford OX1 2JD, United Kingdom;; ^c^Future of Humanity Institute, University of Oxford, Oxford OX1 2JD, United Kingdom;; ^d^Medical Sciences Division, University of Oxford, Oxford OX1 2JD, United Kingdom;; ^e^Department of Computer Science, University of Oxford, Oxford OX1 2JD, United Kingdom;; ^f^Computational Epidemiology Lab, Boston Children’s Hospital, Boston, MA 02215;; ^g^Department of Pediatrics, Harvard Medical School, Boston, MA 02115;; ^h^Oxford Applied and Theoretical Machine Learning Group, Department of Computer Science, University of Oxford, Oxford OX1 2JD, United Kingdom;; ^i^Department of Public Health, University of Copenhagen, 1165 Copenhagen, Denmark;; ^j^Medical Research Council Centre for Global Infectious Disease Analysis, Imperial College London, London SW7 2BX, United Kingdom;; ^k^Department of Statistics, University of Oxford, Oxford OX1 2JD, United Kingdom;; ^l^Department of Engineering Science, University of Oxford, Oxford OX1 2JD, United Kingdom

**Keywords:** COVID-19, epidemiology, Bayesian modeling, hierarchical modeling, face masks

## Abstract

The effectiveness of mask wearing at controlling severe acute respiratory syndrome coronavirus 2 (SARS-CoV-2) transmission has been unclear. While masks are known to substantially reduce disease transmission in healthcare settings [D. K. Chu *et al., Lancet* 395, 1973–1987 (2020); J. Howard *et al., Proc. Natl. Acad. Sci. U.S.A.* 118, e2014564118 (2021); Y. Cheng *et al., Science* eabg6296 (2021)], studies in community settings report inconsistent results [H. M. Ollila *et al.,* medRxiv (2020); J. Brainard *et al., Eurosurveillance* 25, 2000725 (2020); T. Jefferson *et al., Cochrane Database Syst. Rev.* 11, CD006207 (2020)]. Most such studies focus on how masks impact transmission, by analyzing how effective government mask mandates are. However, we find that widespread voluntary mask wearing, and other data limitations, make mandate effectiveness a poor proxy for mask-wearing effectiveness. We directly analyze the effect of mask wearing on SARS-CoV-2 transmission, drawing on several datasets covering 92 regions on six continents, including the largest survey of wearing behavior (n= 20 million) [F. Kreuter *et al.*, https://gisumd.github.io/COVID-19-API-Documentation (2020)]. Using a Bayesian hierarchical model, we estimate the effect of mask wearing on transmission, by linking reported wearing levels to reported cases in each region, while adjusting for mobility and nonpharmaceutical interventions (NPIs), such as bans on large gatherings. Our estimates imply that the mean observed level of mask wearing corresponds to a 19% decrease in the reproduction number *R*. We also assess the robustness of our results in 60 tests spanning 20 sensitivity analyses. In light of these results, policy makers can effectively reduce transmission by intervening to increase mask wearing.

## Significance

We resolve conflicting results regarding mask wearing against COVID-19. Most previous work focused on mask mandates; we study the effect of mask wearing directly. We find that population mask wearing notably reduced SARS-CoV-2 transmission (mean mask-wearing levels corresponding to a 19% decrease in R). We use the largest wearing survey (n = 20 million) and obtain our estimates from regions across six continents. We account for nonpharmaceutical interventions and time spent in public, and quantify our uncertainty. Factors additional to mask mandates influenced the worldwide early uptake of mask wearing. Our analysis goes further than past work in the quality of wearing data–100 times the size with random sampling–geographical scope, a semimechanistic infection model, and the validation of our results.

**Fig. 1.**
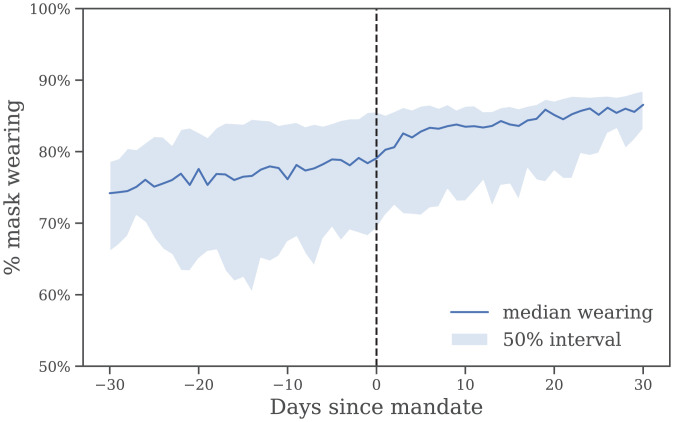
Reported mask wearing in countries with <40% of population fully vaccinated, as of 1 October 2021 [wearing from the UMD/Facebook survey (1); vaccinations from ref. 2]. The *y* axis is the proportion who reported that, over the last week, they wore masks most or all of the time in public spaces.

Face masks are one of the most prominent interventions against COVID-19, with very high uptake in most countries (1). However, global mask wearing fell substantially in 2021, even in countries with low vaccination rates (Fig. 1). Given ongoing epidemics, establishing the effectiveness of mask wearing in community settings is critical. The following sections review past work on the effectiveness of mask wearing in different settings and at different scales.

In the context of healthcare, N95 masks (as defined by ref. 3) work well when worn properly by trained users—reducing transmission of coronaviruses including severe acute respiratory coronavirus syndrome 2 (SARS-CoV-2) by at least half (4, 5). Cheng et al. (6) find that ideal surgical masking (7, 8) of a noninfected person corresponds to a 65 to 75% reduction in their risk of COVID-19.

However, the effect of mask wearing in small-scale community settings is more difficult to detect.

In particular, four meta-analyses have summarized studies on respiratory infections, conducted in community settings (4, 9–11). They estimate mean decreases in infection risk between 4% and 15% for surgical masks, but with large uncertainty: Individual results ranged from a 7% increase in infection risk to a 61% decrease in infection risk. In addition, few of these studies are randomized controlled trials (RCTs), and those that are RCTs have considerable issues: Bungaard et al. (12) found a small, nonsignificant reduction in infection risk. Abaluck et al. (13), found a significant, 8.6% decrease in symptomatic seropositivity linked to mask wearing. However, limitations of the study included a requirement for unblinded participants to self-report symptoms before testing, use of an antibody test with a very low 5 d sensitivity, and unclear generalization from the specific context (rural villages in Bangladesh).

We focus on the effects of mask wearing or mandates (i.e., legal requirements to wear a mask) on transmission in large connected populations. To study mask impacts on transmission, many studies use the timing of mask mandates as a proxy for sharp changes in the level of mask wearing. Some such studies have inferred limited or inconclusive effects in cross-country analyses (14) and within-country studies (15), while others find cross-country evidence that mask mandates and recommendations lead to decreased transmission and mortality (16, 17).

Other analyses provide evidence for reduced case growth following subnational mandates within countries such as the United States (18–20) and Germany (21). A potential explanation for the inconsistency and uncertainty of these results is that data on national mandate timing may be poorly suited for analyzing the effects of mask wearing on transmission.

Epidemiological studies often use government mask mandates as a proxy for mask wearing. However, the existing literature on the relationship between mandates and actual levels of mask wearing has shown surprisingly weak effects. For example, studying US states, ref. 22 failed to find a statistically significant relationship between mandates and subsequent wearing, while other studies found postmandate increases in wearing of just 13% (23) and 23% (24). Betsch et al. (25) find a ∼40% increase in wearing after local mandates in Germany, but no other study finds a comparably large increase. Given that the link between mandates and wearing is surprisingly weak, it is likely that the link between mandates and transmission is difficult to detect. Three additional factors lead us to suspect that a link between mandates and transmission would be difficult to detect. First, introducing a mandate is a coarse, one-off event that necessarily loses signal by not tracking day-to-day changes in mask wearing. We also have fewer data on mandates: Less than half of the regions we study enforced any mandate during the study period. Second, past studies treat mandates as a binary on/off intervention that is fully implemented at a single point in time. However, modeling the effect of mandates as an instantaneous change in the reproduction number or mortality fails to capture changes in wearing behavior following the announcement of a mandate but before its enforcement (21). Nor does it account for gradual change in behavior after the implementation of a mandate. Finally, the circumstances of mandate policies are highly heterogeneous, both in terms of the preexisting level of voluntary wearing at the time of implementation and in terms of how exactly they are defined, enforced, and complied with. Consequently, averaging the international effect of mandates based on coarse data is unlikely to provide a useful summary of heterogeneous mandate effects. Importantly, these arguments point to the link between mandates and transmission being difficult to detect, not that it is absent.

Because of these difficulties in studying the effect of mandates, we instead focus on estimating the effect of mask wearing on transmission, using a large (*n* = 19.97 million) global survey of self-reported mask wearing (1). Two other studies estimate mask effectiveness from self-reports: In their study of 24 countries, Aravindakshan et al. (26) use YouGov wearing data to infer an overall 3.9 to 10% relative decrease in case growth rate from whole population mask wearing. Rader et al. (22) study US states using a novel SurveyMonkey wearing dataset to infer a ∼10% decrease in transmission between the lowest and highest empirical quartiles of wearing (a 50 to 75% increase in wearing). Rader et al. use data limited to 12 US states during June–July 2020. Our data are richer: We study 56 countries on six continents, and our inferential analyses span May–September 2020.

Our analysis goes further than past work in the quality of wearing data—100 times the sample size, with random sampling and poststratification—the geographical scope, the use of a semimechanistic infection model, the incorporation of uncertainty into epidemiological parameters, and the robustness of our results (59 sensitivity tests). See Table 1 for our operational definitions.

**Table 1.** Glossary of key terms

Terminology	Meaning
Clinical settings	Any inpatient setting involving healthcare professionals. These include hospitals, doctor’s offices, and other inpatient clinics; this covers the place, and so includes cleaners and receptionists (and anyone else) who are in contact with patients in inpatient settings. It would not include, for example, administrators working in an office attached to a hospital, or paramedics attending at an emergency.
Community settings	Any setting outside clinical or residential settings, such as public areas, restaurants, and public transportation, as well as public and private indoor areas.
Mask	Any face covering. Unless specified, this is broadly construed to include both cloth and surgical-grade masks and above. See also refs. 3 and 7.
Mask wearing	All community mask wearing: the proportion of people wearing masks in community settings.
Reported mask wearing	The quantity of self-reported wearing in the following sense: Over the last week, respondents wore a mask most or all of the time when in public spaces; a proxy.
Mandate	As per OxCGRT, a legal requirement to wear a mask, in a (usually national) region, “in [at least] some specified shared spaces outside the home with other people present or some situations when social distancing [is] not possible.”
Epidemiological effect	An effect studied at a population level, measured in entire populations, rather than with data observed at the individual level.
NPI	A policy implemented to prevent transmission, excluding pharmaceuticals such as vaccines and therapeutics. Examples include school and business closures, stay-at-home orders, and restrictions on gatherings.

## Results

### The Effect of Mask Wearing on Transmission.

Using data from May to September 2020, we estimate the effects of mask wearing on transmission in 92 regions (*SI Appendix*, Table S3). This window of analysis is determined by our datasets: the University of Maryland (UMD)/Facebook COVID-19 World Symptoms Survey (1, 27) reports wearing estimates for our regions starting from 1 May 2020 (1). We end on 1 September 2020, at the beginning of the second wave, a period in which national nonpharmaceutical interventions (NPIs) fragment into regional responses, making national analyses less informative (16). Our wearing estimates are from UMD and (for the United States) the COVIDNearYou/SurveyMonkey dataset (22). Our covariate “percentage of region wearing masks” is the weighted percentage of people who said that, over the past 7 d, they wore masks in public most or all of the time. The weights correct for nonresponse bias and for demographic imbalance (27). This is a proxy for the true wearing level (see *Discussion*).

**Fig. 2.**
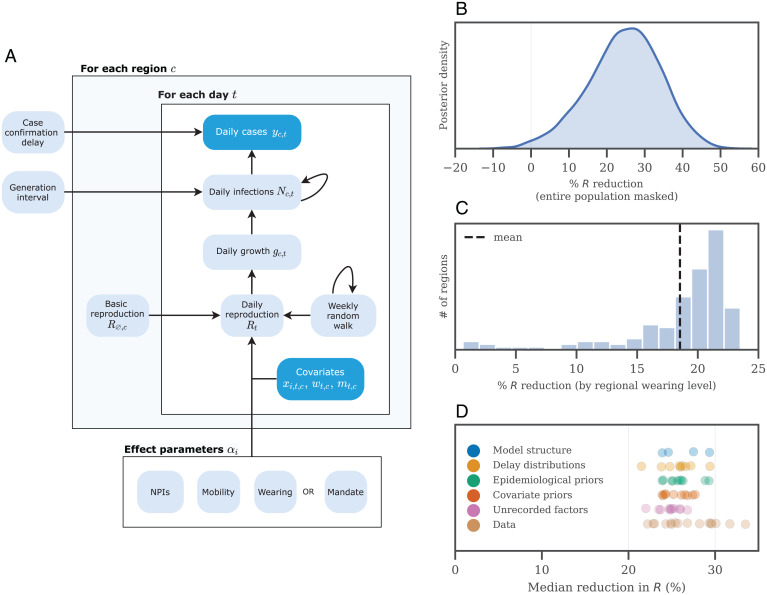
(*A*) (*Top*) Model schematic. Observed nodes are dark blue, latent nodes are light blue. (*Bottom*) The target of our analysis is *α_i_*, which includes NPIs, mobility, and masks, and is assumed to be the same for each country (as we do not have enough data to estimate country-specific effects). On each day *t*, region *c*’s reproduction number *R_t_* depends on 1) the starting reproduction number $R_{\text{init},c}$, 2) the NPIs active in region *c*, 3) the mobility level, 4) either the wearing level or the mandate indicator, and 5) a location-specific weekly random walk. The resulting *R_t_* estimate (as a growth rate) is used to compute the latent daily infections *N_t_*, given the distributions over the generation interval and the previous infection count. The expected number of daily confirmed cases (*y_t_*) is computed using *N_t_* and the distribution over the delay until case confirmation. (*B*) Posterior reduction in *R* if self-reported wearing increased from 0 to 100%, estimated from all countries. (*C*) Posterior mean estimates for the achieved reduction in *R* from masking in each of our 92 regions (the mean from *B* multiplied by time-averaged wearing in each region). (*D*) Wearing effect estimates over all sensitivity tests; each dot is the median under a different experimental condition (effect on transmission of 100% self-reported wearing).

We use a Bayesian hierarchical model (Fig. 2*A*). The model links wearing levels to the number of reported cases in each region, via the instantaneous reproduction number *R_t_*. Accordingly, our model captures the natural, nonlinear exponential growing or decaying nature of epidemics. Our model is similar to ref. 28, but, in addition to adjusting for other NPIs, we also account for changes in mobility. (See *Data* below for explanations of NPIs and the mobility proxy.) We model many sources of uncertainty through prior distributions: epidemiological properties of the virus, differences in transmission between regions, the lag between an infection and the registration of a COVID-19 case, and the effect of unobserved influences on *R*. Our model shares information across all regions to produce a statistically robust estimate, and thus measures the international mass wearing effect.

Fig. 2*B* shows the inferred wearing effect in the form of a percentage reduction in *R*. We find that the difference between zero mask wearing and 100% of people self-reporting that they mask most of the time in some public places corresponds to a 25% [6%, 43%] reduction in transmission.

In practice, 100% mask wearing is not achievable. Indeed, the maximum possible level of wearing in a region will depend on complex social and cultural factors. To capture these differences, Fig. 2*C* shows our median wearing effect estimate (i.e., the median of the posterior of Fig. 2*B*) multiplied by the median (time-averaged) wearing percentage in each region. Across this window, the mean region saw an average reduction in transmission from wearing of 19%.

### The Effect of Mask Mandates on Transmission.

We illustrate the difficulty of directly inferring how mandates affect transmission, by running our model using mandate data in place of wearing data (*SI Appendix*, section B). We model mask mandates as having an instantaneous effect on wearing (and hence on *R*), a gradually increasing effect, or an effect starting when the mandate is announced but not yet implemented.

**Fig. 3.**
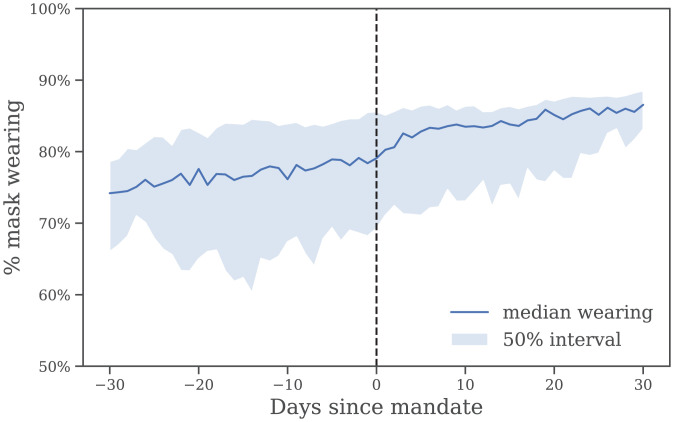
Self-reported mask wearing against mandate timing, averaged over all regions with a new national mask mandate, May–September 2020. Dashed line is the date the mandate began to be enforced.

Each of these models fails to discover a mandate-driven effect on *R*. Presumably, this is because the issue is not one of timing, but that mandates are coarse, heterogeneous, and increase wearing by an average of only 8.6% in our data (Fig. 3). However, our results do provide evidence that mass mask wearing substantially reduces transmission.

### The Mandate–Wearing Correlation.

We can investigate the relationship between mask mandates and subsequent changes in wearing in our data. Fig. 3 shows the wearing trend before and after the implementation of national mandates, averaged across regions. In this context, a mandate implementation date refers to the date when masks were “required in some or all shared spaces, outside the home with other people present, or some situations when social distancing [was] not possible” (29). (See *SI Appendix*, Table S4 for implementation dates.)

Crucially, most of the uptake in wearing occurs premandate.

**Fig. 4.**
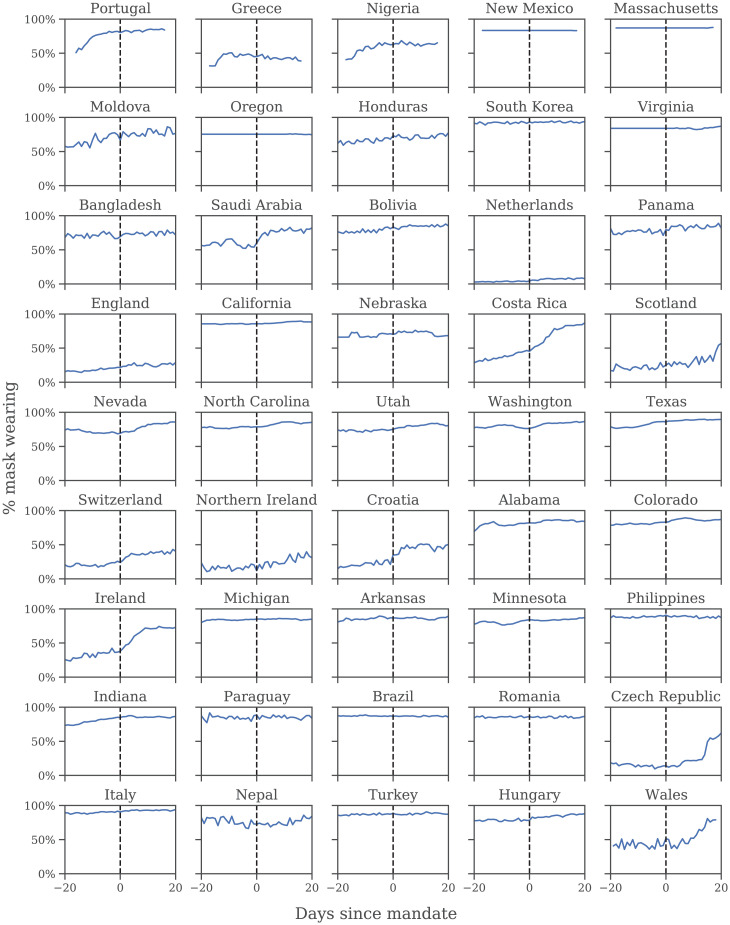
Self-reported mask wearing against mandate timing in all regions with a new national mask mandate, May–September 2020. Dashed line is the date each mandate began to be enforced. Ordered by mandate date; see *SI Appendix*, Table S4.

Fig. 4 illustrates several ways mandates can fail to correlate with wearing: South Korea’s mandate came after voluntary wearing had already plateaued at 94%; conversely, in the Netherlands and Switzerland—which imposed limited mandates for public transport—few people reported wearing masks most of the time, even 3 wk into the mandate period; finally, in the Czech Republic, wearing increased, but only long after the mandate was implemented. On the other hand, a strong correlation between mandates and wearing was observed in Ireland (Fig. 4) and in Germany’s April 2020 local mask mandates (21, 25).

Our sources of wearing data begin after April 2020—that is, after the initial transition to mask wearing in some regions. Since it is possible that earlier mandates had persistent effects on wearing, we investigate the correlation during the first wave using an earlier YouGov wearing survey (*SI Appendix*, section A). In regions with available data, most of the increase in mask wearing occurred before the earliest national government mandates, with 64% average wearing on the day the mandate was enacted and 75% 3 wk following the mandate. However, assessing the true correlation with the available data are difficult—see *Discussion*.

There are several limitations to this analysis: Our wearing data are a proxy for true levels of wearing, and additionally do not capture changes in the quality of mask use, or the heterogeneity in mask type or venue. This is of particular concern at high levels of self-reported wearing: At 100% self-reported wearing, any additional benefit in wearing (quality of mask, frequency of use, and venue) is impossible to capture with these data. Moreover, it is difficult to untangle voluntary wearing from wearing caused by mandates.

## Robustness

Results that are sensitive to plausible alternative model assumptions offer weak evidence and pose a risk of misinforming policy. Therefore, we verify the robustness of our results by performing 60 tests across 20 sensitivity analyses (*SI Appendix*, Table S5). Fig. 2*D* shows how the median effect of wearing changes as we vary epidemiological priors, delay distributions, covariate effect priors, the model structure, and the data. Each point in Fig. 2*D* is the median effect of a different experimental condition. Our results are robust to these changes—95% of the median reductions fall between 22.7% and 31.3%.

However, as this study is observational, caution is necessary when making causal interpretations. Unobserved factors may influence *R*, and, if their timing coincides with the timing of mask wearing, reductions in *R* from unobserved factors may be wrongly attributed to mask wearing (30)—our observed factors will be confounded. For instance, nonwearing protective behaviors like social distancing may potentially confound our estimates, although some will be partially captured by our accounting for changes in mobility (4, 25). We investigate the susceptibility of our results to such confounding in four sensitivity analyses. In the first three (*SI Appendix*, Figs. S15, S16, and S18), we assess how much our estimates change when we exclude previously observed factors: We exclude each NPI in turn, all NPIs at the same time, and the mobility covariate. The small difference between our adjusted and unadjusted estimates suggests that, unless the confounding from unobserved factors greatly exceeds the confounding from our previously observed factors (NPIs and mobility), our results are unlikely to be meaningfully affected by confounding (31). Lastly, over our window of analysis, mask wearing increases while transmission decreases (in many regions). Our final analysis aims to assess whether this correlation is a spurious contributor to the substantial apparent wearing effect. We test this hypothesis by creating a fake wearing variable for each region. Each variable has the same start and end wearing value as the true wearing percentage and linearly interpolates between these values to capture the trend in wearing in that region. We infer a small and uncertain effect for the fake wearing variable 7.6% (–20.2%, 30.0%) (*SI Appendix*, Fig. S17). This implies that the wearing effect we infer does not rely solely on the correlation between transmission and the overall wearing trend in this period.

Another concern for observational NPI studies is endogeneity: When cases are rising, people are more likely to voluntarily mask, and governments are more likely to mandate wearing (32). However, in our window, the correlation between new cases and mask-wearing percentage is low, Spearman’s ρ=0.05, which limits the scope of this concern.

## Discussion

Using several datasets from 92 regions and a state-of-the-art Bayesian hierarchical model, we find evidence that mask wearing is associated with a notable reduction in SARS-CoV-2 transmission. Our analysis adjusts for both NPIs and mobility, and the results are robust to extensive sensitivity analyses. Our analysis of the mandate–wearing correlation suggests that factors beyond mandates strongly affect wearing levels—but does not imply that mandates have no role in curtailing transmission. Instead, the evidence that mass mask wearing reduces transmission implies that mandates (and other mask-promotion policies) may be effective against COVID-19 if and when they improve or increase the use of masks.

### Heterogeneity.

In the Introduction, we highlighted the inconclusive epidemiological literature. This is, in part, due to not accounting for factors relating to mask properties and wearing behavior. These factors include mask quality (33), mask fit (33), the venue of wearing (e.g., in shops, schools, or public transport) (33), mask reuse (34), risk compensation (35), and cultural norms (17, 33, 35). More research into these factors is required to further reduce our uncertainty about mask-wearing effects. We estimate the effect of mass mask wearing, aggregating over mask properties and behavior. Given that, in this window, most masks in use were the least-effective types (cloth or otherwise unrated masks) (4, 34, 36–38), the actual effectiveness of mass wearing today is likely stronger than we estimate.

Masks have at least two effects: preventing transmission to noninfected mask wearers (“wearer protection”) and preventing infected wearers from infecting others (“source control”). With the exception of refs. 6 and 13, the studies listed in the Introduction estimate individual wearer protection, rather than the most policy-relevant quantity: the effect of mass mask wearing including all relevant factors (5, 6, 39).

Additionally, clinical studies may not reflect the actual distribution of protection: For instance, few studies include cloth masks, one of the most common types (37, 38). Finally, while mask wearing is known to be strongly mediated by cultural factors (17, 25, 40), most studies are conducted in a specific social context and may have limited external validity.

### Window of Analysis.

Our results are based on the period from May to September 2020. While we find similar results for different (shorter) windows of analysis (*SI Appendix*, Fig. S34), mass wearing effectiveness will likely differ with larger changes in circumstances. In particular, our period has features that may not characterize other settings; for example, summer months are thought to have lower transmission (41, 42), and a tiered regional approach to containment was not yet implemented in most regions. However, a short window implicitly holds many factors constant. This is useful for internal validity: When estimating a specific quantity such as the effects of mask wearing, a short window reduces the scope for distribution shift and unobserved confounders.

### Operationalizing Mask Wearing.

Importantly, our effect estimates rely on self-reports of mask wearing from surveys, which are ultimately a proxy for actual wearing behavior. Social desirability bias may inflate wearing estimates (43): in one Kenyan study, the disparity between self-reported wearing and observed wearing was 77% (44)—although this survey was not anonymous, which may have led to more overreporting than anonymous surveys such as COVIDNearYou–SurveyMonkey. If data sources overestimate mask wearing, then our estimate for the effect of 100% of people wearing masks (most or all of the time) will actually correspond to the effect of less than 100% of people wearing masks. Consequently, we would expect the true effect of 100% mask wearing to be larger than we estimate, in proportion to the amount of overreporting. Further, the operational definition of “mask wearing” used in the UMD survey is not stringent: It can be applied both to a person who wears a cloth mask, only on public transport, slightly more than half of the time, and to a person who always wears an N95 respirator when outside their home (1). This implies that there is probably scope for more and better mask wearing, even in regions reporting, in our data, extremely high levels of wearing.

### Conclusion.

We find that mask wearing is associated with a notable reduction in transmission. Our evidence shows that factors other than mandates must have contributed to the worldwide uptake of mask wearing in 2020. In situations where mandates are unlikely to have a large effect on uptake—for example, because voluntary wearing is already high—policy makers may be able to use other levers to increase wearing quantity and quality. For example, if masks are widely used but are often of poor quality, or worn incorrectly, or are not worn in the most important venues, then policy makers can respond with education about correct mask fitting and quality, as well as mandates that focus on venues with the greatest risk of transmission (5, 45).

## Materials and Methods

All data and code used can be downloaded via Zenodo: https://zenodo.org/record/6385347#.Yk9Ufi-B3vw. The preprocessing is derived from ref. 28.

### Data.

Our analysis is on the national (or US state) level, since this is the finest resolution available for all countries in the Oxford COVID-19 Government Response Tracker (OxCGRT) NPI dataset. OxCGRT represents each NPI policy as a binary variable (“Is this policy active throughout this region at time *t*, or only in certain locales?”) along with an intensity variable marking how strict the implementation was. Table 2 summarizes the modeling set.

**Table 2.** Modeling data summary

Category	Data
Regions	92 (55 countries + 37 US states)
Period	1 May 2020 to 1 September 2020
Modeling data points	13,248 d across all regions
Mask wearing data points	19.97m [UMD (1)] + 558,670 [COVIDNearYou (22)]
Case data	JHU CSSE dataset (46)
Additional data	Google mobility (47); OxCGRT NPIs (29)
Data validation	Manual correction of reporting errors; filtering out nonepidemic regions; validation against external sources

Daily national estimates of mask wearing are derived from the UMD/Facebook COVID-19 World Symptoms Survey (1), which randomly samples from all active Facebook users, and which poststratifies to correct for nonresponse bias and demographic imbalance (27). The mean number of individual responses per region-day is 1,131. UMD does not cover the United States, so we supplement this dataset with the US data of ref. 22, which, in our window, represent *n* = 558,670 responses.

Daily confirmed COVID-19 cases are drawn from the Johns Hopkins Center for Systems Science and Engineering (CSSE) COVID-19 Data Repository, which collates official statistics from around the world (46). Dates are standardized to the date the case was initially reported to the official body. Unfortunately, cases by specimen date are not available for the majority of the countries in our sample; this is why international case databases (e.g., John Hopkins, European Centre for Disease Prevention and Control) exclusively use the date of reporting, to make case numbers more consistent between countries.

By “mobility,” we mean how much public activity there is in a region. As a proxy, we use the Google COVID-19 Community Mobility Reports (47), which tracks the presence of Android smartphones in particular sectors of society (commercial, residential, parks), and relates the current level of activity to the 2019 prepandemic level. It is thus a proxy and a relative measure, but one which has been useful for tracking voluntary safety measures (48, 49). We take the average of the commercial and workplace mobility indices, because these are the sectors with the most risk.

See *SI Appendix*, section A for full data details, including preprocessing steps and region selection.

### Model.

We develop a hierarchical Bayesian model based on prior work (16, 28, 50) to infer the effectiveness of mask wearing on COVID-19 transmission (Fig. 2*A*). That is, we construct a probabilistic model with prior distributions over parameters and hyperparameters, and use Markov Chain Monte Carlo sampling to produce a posterior estimate representing our full uncertainty over the target effect (51). We use the number of reported cases in each country to infer the number of later-ascertained infections on each day. Given the dynamics of daily, later-ascertained infections in each region over time, we infer the instantaneous reproduction number *R_t_*. Finally, the covariate effects are estimated by relating the *R_t_* to the observed level of each covariate. The Bayesian approach allows us to explicitly model sources of uncertainty, such as uncertain values of epidemiological parameters.

We now outline the inputs of our model.

#### Notation.

We use *c* to denote the country/region in question, and use *t* to index time; *t* = 0 corresponds to 1 May 2020. NPIs are indexed by *i*.

#### Inputs.

• **NPIs**: $x_{i,t,c}\in \{0,1\}$. $x_{i,t,c}=1$ if NPI *i* is active at time *t* in region *c*; otherwise, $x_{i,t,c}=0$.
• **NPI reopenings**: NPIs were active in many regions at the start of our period. We treat these NPIs, in the relevant regions, as “reopening” NPIs. If NPI *i* is active in region *c* at *t* = 0 (i.e., we have $x_{i,0,c}=1$), we subtract one from the feature to form ${\bar{x}}_{i,t,c}$. Therefore, at the start of the window, ${\bar{x}}_{i,t,c}=0$, and the effect of the NPI is absorbed into $R_{\text{init},c}$. When the NPI lifts, we would have ${\bar{x}}_{i,t,c}=-1$, reflecting that NPI lifting has the opposite effect from NPI closing, which is denoted as $x_{i,t,c}=1$. As such, we can more easily set a prior over $R_{\text{init},c}$ (see *Prior Distributions*, below).
  [1] $${\bar{x}}_{i,t,c}=\{\begin{array}{ll} x_{i,t,c}-1 & \text{if}\;x_{i,0,c}=1,\\ x_{i,t,c} & \text{otherwise} \end{array}.$$
• **Mask wearing**: The percentage of people in each region that self-report as likely to/always wear masks in public, $w_{t,c}\in [0,1]$.
• **Mobility**: Reduction in mobility relative to 2019 levels $m_{t,c}$, represented as a multiplicative factor.
  $$m_{t,c}=({\text{mobility}}_{2019}-{\text{mobility}}_{t,c})\;/\;{\text{mobility}}_{2019},$$
  where $m_{t,c}=1$ represents a 100% decrease in mobility, while $m_{t,c}=0$ represents no change from 2019 level.
• **Cases**: New confirmed cases observed on day *t*: $y_{t,c}$.

In the following sections, we introduce several variables without explicitly defining them. They are defined in *Prior Distributions*, below.

#### Infection Model.

The instantaneous reproduction number $R_{t,c}$ is the expected number of infections that would arise from each infection at time *t* in region *c*, all else equal. We model *R_t_* as a product of several terms: 1) the regional starting reproduction number $R_{\text{init},c}$; 2) a product of our effect estimates for that region-day for each of the reopening NPIs $X_{t,c}$, mask wearing $W_{t,c}$, and mobility $M{\left(m_{c,t}\right)}^{-}$; and 3) a weekly latent random walk per region $z_{t,c}$.

[2] $$R_{t,c}=R_{\text{init},c}\cdot X_{t,c}\cdot W_{t,c}\cdot M_{t,c}^{-}\cdot \exp\;(z_{t,c}).$$

We will now discuss each of these terms in turn.

##### Latent reproduction number.

The latent, unobserved reproduction number in region *c* at *t* = 0, assuming no mask wearing, is represented by $R_{\text{init},c}$.

##### NPIs.

We assume that the introduction or lifting of an NPI leads to an instantaneous, multiplicative change in transmission. Each NPI contributes $\exp\;(-\alpha_{i}\cdot {\bar{x}}_{i,t,c})$ to $R_{t,c}$. Note that this also works for reopening NPIs—if the NPI effect (*α_i_*) is positive, a reopening ($x_{i,t,c}^{-}=-1$) increases *R*,

$$X_{t,c}=\exp\;\left(-\sum_{i=1}^{I}\alpha_{i}\cdot {\bar{x}}_{i,t,c}\right).$$

##### Mask wearing.

$W_{t,c}=\exp\;\left(-\alpha_{w}w_{t,c}\right)$. We use the exponential form in our base model. However, we test the sensitivity of our results to two alternative mask-wearing parameterizations and find similar results (*SI Appendix*, section D, Model structure).

##### Mobility.

We parameterize the Google mobility data as in ref. 48,

$$M(m)=\frac{2\exp\;(-\alpha_{m}m)}{1+\exp\;(-\alpha_{m}m)}.$$

At 2019 levels of mobility (*m* = 0), the multiplicative factor *M*(*m*) = 1, leading to no effect on *R_t_*. To set a principled prior for $R_{\text{init},c}$, we normalize mobility by the initial level (see *Prior Distributions*),

$$M^{-}(m_{c,t})=\frac{M(m_{c,t})}{M(m_{c,0})}.$$

##### Random walk.

The weekly random walk is computed as

$$z_{t,c}\;=\{\begin{array}{ll} 0\; & t\leq 13\\ z_{t-1,c}+\varepsilon_{f(t),c}\; & \text{if}\;t\;\mathrm{mod}\;\;7=0\\ z_{t-1,c} & \text{otherwise} \end{array},$$

where f(t)=⌊(t−14)/7⌋ and $\varepsilon \approx \mathrm{Normal}(0,\sigma_{\text{RW}})$. The random walk starts after 2 wk to avoid unidentifiability between $R_{\text{init},c}$ and the random walk terms at the beginning of the period.

Following ref. 52, the resulting *R_t_* estimate is then transformed to daily growth using the generation interval distribution, which describes the time between successive infection events in a transmission chain. $N_{t,c}$ represents daily infections that are later ascertained, and we have $N_{t,c}=g_{t-1,c}\cdot N_{t-1,c}$; that is, we multiply the infections on the previous day by the daily growth rate. Then, given an initial (latent) infection count, we have

$$\begin{array}{l} \beta_{\text{GI}}=\frac{\mu_{\text{GI}}}{\sigma_{\text{GI}}^{2}},\;\alpha_{\text{GI}}=\frac{\mu_{\text{GI}}^{2}}{\sigma_{\text{GI}}^{2}},\\ g_{t,c}=\beta_{\text{GI}}\left[\exp\;\left(\frac{R_{t,c}}{\alpha_{\text{GI}}}\right)-1\right].\\ N_{t,c}=N_{0,c}\prod_{\tau =1}^{t}\left(1+g_{\tau ,c}\right) \end{array}$$

#### Observation model.

Infections at time *t* are only observed as reported cases after a delay. Therefore, we convolve the later-ascertained cases with a delay vector to produce ${\bar{y}}_{t,c}$, which is the expected number of reported cases on day *t* in country *c*.

$${\bar{y}}_{t,c}=\sum_{\tau =0}^{31}N_{t-\tau ,c}\cdot T[\tau ].$$

The forward-delay vector T (defined in *Prior Distributions*, below) defines the delay between the two quantities. Finally, the observed number of reported cases, $y_{t,c}$, follows a negative binomial distribution,

[3] $$y_{t,c}\approx \mathrm{NegBin}(\mu ={\bar{y}}_{t,c},\alpha =\Psi ),$$

where Ψ is the case-reporting overdispersion parameter (see below).

#### Prior distributions.

We place prior and hyperprior (prior distributions placed on parameters describing another prior) distributions over several parameters. Our Bayesian approach not only captures uncertainty in unknown parameters but allows our beliefs about certain parameters to be adjusted if warranted by the data. We now detail the priors we use in this work.

• **Region-specific initial reproduction number**: $R_{\text{init},c}\approx \;\mathrm{Normal}(\mu_{R},\sigma_{R});$
• $R_{\text{init}}$ **hyperpriors**: the Epidemic Forecasting group (53) produced estimates for $R_{t,c}$ using methodology from ref. 54. The empirical mean and variability of these estimates across our regions at the start of our period is μ​=​1.07,σ​=​0.32. We use these estimates to initialize our hyperpriors over the mean and variability of $R_{\text{init},c}$,
  $$\begin{array}{l} \mu_{R}=\mathrm{TruncatedNormal}(\mu \text{​}=\text{​}1.07,\sigma \text{​}=\text{​}0.2,\mathrm{lower}\text{​}=\text{​}0.1),\\ \sigma_{R}=\mathrm{HalfNormal}(\sigma \text{​}=\text{​}0.4).\;\text{Median​}=\text{​}0.32. \end{array}$$
• **NPI effect**: $\alpha_{i}\text{​}\approx \text{​}\mathrm{AsymmetricLaplace}(m\text{​}=\text{​}0,\kappa \text{​}=\text{​}0.5,\lambda \text{​}=\text{​}30)$, following ref. 28. Here *m* is the location, *κ* is the asymmetry, and *λ* is the scale. This prior places 80% of its mass on positive NPI effects (i.e., on reductions of *R*).
• **Wearing effect**: $\alpha_{w}\text{​}\approx \text{​}\mathrm{Normal}(\mu \text{​}=\text{​}0,\sigma \text{​}=\text{​}0.4)$. Unlike the NPIs above, the prior for wearing has equal mass on positive and negative effects. This uninformative choice reflects past uncertainty about the efficacy of mask wearing.
• **Mobility effect**: $\alpha_{m}\text{​}\approx \text{​}\mathrm{Normal}(\mu \text{​}=\text{​}1.704,\sigma \text{​}=\text{​}0.44)$. Mobility prior values are derived from the “overall average mobility” estimate in ref. 48.
  Note that each *α* above is not a direct reduction in *R*; they are transformed into a reduction via a specific functional form (see above).
• **Initial infection counts:** Initialized with the empirical median new confirmed cases of the first day of our window, $\log\;{\tilde{y}}_{0}=5.46$.
  $$\begin{array}{l} \zeta_{c}\approx \mathrm{Normal}(\mu \text{​}=\text{​}5.46,\sigma \text{​}=\text{​}5.46)\\ N_{0,c}=\exp\;(\zeta_{c}). \end{array}$$
• **Random walk noise scale**, chosen as in ref. 16,
  [4] $$\sigma_{RW}=\mathrm{HalfNormal}(\mu \text{​}=\text{​}0,\sigma \text{​}=\text{​}0.15).$$
• **Generation interval distribution** (55, 56):
  $$\begin{array}{l} \mu_{\text{GI}}\approx \mathrm{Normal}(\mu \text{​}=\text{​}5.06,\sigma \text{​}=\text{​}0.33),\\ \sigma_{\text{GI}}\approx \mathrm{Normal}(\mu \text{​}=\text{​}2.11,\sigma \text{​}=\text{​}0.5). \end{array}$$
• **Time from infection to case confirmation** T (28, 56–58): The delay between infection and case confirmation is distributed as
  D≈NegBin(μ​=​10.92,α​=​5.41).
  We produce a forward-delay vector
  $$\begin{array}{l} T[t]=\{\begin{array}{ll} \frac{1}{Z_{C}}D(t)\; & t<32\\ 0 & \text{otherwise} \end{array},\\ \text{with}\;Z_{C}=\sum_{t\prime =0}^{31}D(t\prime ), \end{array}$$
  that is, a negative binomial distribution, truncated at 31 d and normalized. Note that the negative binomial *α* parameter denotes the dispersion, not the variance, $\sigma^{2}=\mu +(\mu^{2}/\alpha )$.
• **Observation noise dispersion**, chosen as in ref. 28,
  [5] Ψ≈HalfNormal(μ​=​0,σ​=​5).

## Supplementary Material

Supplementary File

## Data Availability

Previously published data were used for this work (1, 22, 29, 46, 47, 59). Open data and code to obtain and process these datasets are available at Zenodo, https://zenodo.org/record/6385347#.Yk9Ufi-B3vw.
