# Supplementary material for: Mask wearing in community settings reduces SARS-CoV-2 transmission
Source: Proc Natl Acad Sci U S A. 2022 May 31;119(23):e2119266119. doi: 10.1073/pnas.2119266119 (PMC9191667; doi:10.1073/pnas.2119266119)
Supplement: Supplementary File [file pnas.2119266119.sapp.pdf]

Supplementary Information

A. Data

JHU CSSE cases database. We take daily confirmed case counts from the Johns Hopkins University Center for Systems Science and Engineering COVID-19 Global Cases dataset (1), which collates official statistics from hundreds of world regions. We use confirmed cases, because the alternative response variables have worse data quality or availability. In our window, deaths are low in most regions: in fact, 28% of region-days have 0 deaths; they also tend to show severe periodicity in this window; and death data have their own ascertainment issues (e.g. changing rates of post-mortem testing throughout the epidemic). To our knowledge, daily hospitalisations are only available for 15 of our 55 countries (2). Lastly, excess mortality is rarely available at the required granularity, and it also represents many causes of death besides COVID (e.g. decreased flu mortality, traffic mortality, etc).

Many countries fail to report case numbers over the weekend (or report weekly), which leads to spurious periodicity. In addition, severe reporting errors (day-to-day spikes of 1000% or troughs of less than 10% in countries with hundreds or thousands of daily cases) occur in 23 regions. We manually mask these errors (Table S.1), preventing the model from learning from those days.

**Costa Rica**	2020-09-20, 2020-09-21
**Ethiopia**	2020-06-30, 2020-07-01 to 2020-07-08
**Guatemala**	2020-07-18
**Lebanon**	2020-08-04, 2020-08-05
**Libya**	2020-08-23, 2020-08-24
**Michigan**	2020-08-21, 2020-08-22, 2020-08-29, 2020-08-30
**United Kingdom**	2020-07-01, 2020-07-02
**Honduras**	2020-05-20 to 2020-05-24, 2020-05-29, 2020-05-30
**Netherlands**	2020-08-11, 2020-08-12
**Panama**	2020-06-14, 2020-06-15
**Singapore**	2020-08-05
**Serbia**	2020-07-25, 2020-07-26
**Alabama**	2020-06-27, 2020-06-28
**Arizona**	2020-06-29
**Colorado**	2020-09-04, 2020-09-05
**Delaware**	2020-05-23, 2020-05-24
**Minnesota**	2020-07-04
**New Mexico**	2020-05-23, 2020-05-24
**Oregon**	2020-06-06 to 2020-06-08, 2020-06-13, 2020-06-14
**South Carolina**	2020-06-03 to 2020-06-07
**Washington**	2020-05-23, 2020-05-24
**Wisconsin**	2020-08-18, 2020-08-19
**Iowa**	2020-08-27

Table S.1. Dates of reporting errors in the JHU case data

The OxCGRT NPI database. We take NPI data from the Oxford COVID-19 Government Response Tracker, which collects data at the national-level and US state-level (3). From these we select the 'containment' policies, i.e. direct attempts to reduce transmission.

Importantly, OxCGRT cannot be used for national modelling without imputation. OxCGRT reports only one value per region-day, even if policies differ between regions. The dataset reports the maximum stringency of each NPI, whether or not this is implemented in all regions. This leads to the national stringency value being "hidden" behind the highest regional value, where any region has stronger measures. As a result, when a policy is strengthened in only part of a region, we impute the previous national value.

We process the NPI data as follows:

- We filter to rows with national coverage (Flag columns = 1).
- We threshold the ordinal values as in Table S.2, creating a feature for the first mandatory level of each policy and additional features for higher levels of school closing, workplace closing and restrictions on gatherings. This yields 10 NPI features.
- When a policy is strengthened in only part of a region, we impute the previous national value.

Original feature name	Original scale	Cutoff
C1_School closing	Ordinal (0-3)	2,3
C2_Workplace closing	Ordinal (0-3)	2,3
C3_Cancel public events	Ordinal (0-2)	Obsoleted
C4_Restrictions on gatherings	Ordinal (0-4)	2, 3, 4
C6_Stay at home requirements	Ordinal (0-3)	2
C7_Restrictions on internal movement	Ordinal (0-2)	2
H6_Facial Coverings	Ordinal (0-4)	2, 3
*_Flag	Binary	-

Table S.2. OxCGRT NPI features and our threshold choices

UMD / Facebook wearing dataset. We use the University of Maryland Centre for Geospatial Information Science—Facebook Research survey as our main source of daily, self-reported wearing data (4, 5). This is by far the largest-scale survey of COVID mask-wearing (with 19.97 million individual responses in our window, or 1,500 individual responses per region-day). The survey uses stratified random sampling of all active Facebook users to ensure demographic balance in each region, and also guarantees at most one response per month per Facebook user.

An alternative survey, the Imperial College London—YouGov COVID-19 Behaviour Tracker (6) is one hundred times smaller than UMD, uses nonrandom sampling, and has most days missing, and is as such less suitable for modelling.

We validate UMD against YouGov and the UK Office for National Statistics (7) in Figure S.1. The survey questions:

- UMD: "did you, over the past 7 days, wear masks in public most or all of the time?"
- ONS: "In the past seven days, have you used a face covering when outside your home?"
- YouGov: "% of people reporting wearing a mask when in public places."

We see close agreement between UMD and YouGov, with a higher estimate from the ONS owing to their looser definition.

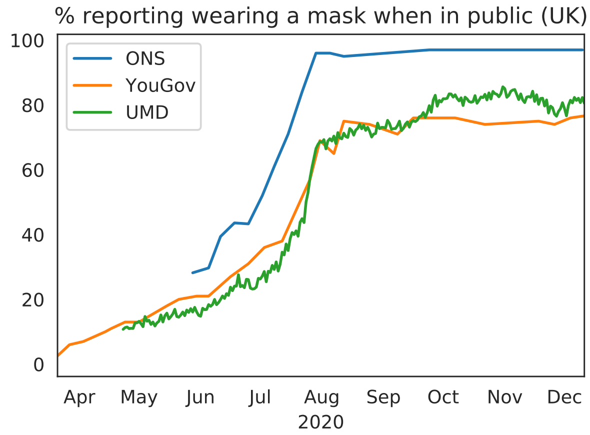

Fig. S.1. Three sources of self-reported wearing data for the UK.

Continent	Region
Asia	Bangladesh, India, Indonesia, Iraq, Israel, Japan, Lebanon, Nepal, Philippines, Saudi Arabia, South Korea, Singapore, United Arab Emirates
Europe	Austria, Belarus, Croatia, Czech Republic, Germany, Greece, Hungary, Ireland, Italy, Moldova, Netherlands, Norway, Poland, Portugal, Romania, Russia, Sweden, Turkey, Ukraine, Switzerland, United Kingdom
Africa	Egypt, Morocco, Libya, Kenya, Nigeria, South Africa
South & Central America	Argentina, Bolivia, Brazil, Colombia, Costa Rica, Dominican Republic, El Salvador, Guatemala, Honduras Mexico, Panama, Paraguay, Venezuela
North America	Canada, Alaska, Alabama, Arkansas, Arizona, California, Colorado, Delaware, Florida, Georgia, Hawaii, Iowa, Illinois, Indiana, Massachusetts, Maryland, Michigan, Minnesota, Missouri, Montana, North Carolina, North Dakota, Nebraska, New Jersey, New Mexico, Nevada, New York, Ohio, Oklahoma, Oregon, Pennsylvania, South Carolina, South Dakota, Texas, Utah, Virginia, Washington, Wisconsin
Oceania	Australia

Table S.3. Regions included in the analysis, by continent

The COVIDNearYou / SurveyMonkey United States wearing dataset. The UMD dataset does not include US wearing data, while the respective CMU / Facebook US survey (8) does not begin reporting until after our window of analysis. We supplement UMD with data from Rader *et al.* (9).

The Rader data are individual survey responses on a reverse Likert scale, weighted to correct for demographic imbalance in the sample. To convert this to the UMD scale, we take the mean of the grocery shopping and workplace features, threshold at ≤ 2 (likely or very likely) and take the percentage of rows in each state passing this threshold, and smooth over a 7 day window. This results in a percentage-wearing feature which is within 1% of the Facebook US data (8) for the period where the two datasets overlap.

The wearing level and new daily cases (1 week moving average) of all regions are plotted in Figure S.16. See also Fig S.14 for a full picture of covariates by region.

Google Mobility Index. We use the Google COVID-19 Community Mobility Reports to index mobility changes in each region (10). We form a single feature by averaging the indoor public components (retail and recreation, grocery and pharmacy, transit, and workplaces). We parameterise mobility similarly to Unwin *et al.* (11).

Instantaneous reproduction number estimates. To validate our model estimates, and for the initialisation of R_0, we use regional R_t estimates from the Epidemic Forecasting group (12). The estimates are calculated using a nonparametric approach from (13). US state-level estimates are taken from https://rt.live/.

Region selection. The OxCGRT dataset has 184 countries, or 235 regions counting US territories. 81 of these regions are missing from the UMD wearing data, and are thus dropped when joining to OxCGRT. We manually drop 32 regions with frequent extreme periodicity in case reporting, 16 that have fewer than 5000 cumulative cases in our window, 10 not contained in the Google Mobility dataset, and 4 that are missing more than 3 consecutive weeks of wearing data. The resulting included regions are shown in Table S.3.

Mask-wearing and mask mandates in the first wave. The YouGov survey (6) begins in Jan 2020 for some locations, which enables us to check the mandate-wearing relationship in the first wave, at the time of the earliest mandates. Figure S.2 displays the estimates against mandate date (including some regions with multiple mandates). The average reported level of mask-wearing in Jan 2020 was 32.7%. This increased to an average of 64.2% before the first national mandate implementations in March and April. There was an average post-mandate increase in wearing of 11%, similar to in our modelling set (an 8.3% post-mandate increase).

Mask recommendations. We follow past work in timing mandates with the beginning of the nominal legal enforcement of wearing. Our source of NPI data (3) also contains an indicator for whether a non-mandatory government recommendation to wear masks was in place. To see if this less stringent, but generally earlier, policy has stronger correlations with subsequent mask-wearing, we repeat the exploratory analysis from above. The correlation between wearing percentage and any form of recommendation or mandate is weaker than before, Spearman's $\rho = 0.23, p < 0.001$, compared to the mandate correlation of 0.32.

Case ascertainment rate. A model that only uses confirmed cases could result in systematically biased estimates if the case ascertainment rate (the ratio of detected cases to actual infections) changes over the window of analysis.

Our model does not use cases naively. As regards the multi-week confirmation delay, the model places a prior over this (centred on 11 days with standard deviation 6 days), and infers a time-varying reporting delay $\mathcal{T}[t]$. Coupled with the noise on R, this allows us to capture changing ascertainment and similar confounders. The model is designed to be able to distinguish changes in the ascertainment rate (AR) from changes in NPIs (including wearing). Our noise terms can capture changes in AR (and other latent quantities, like the

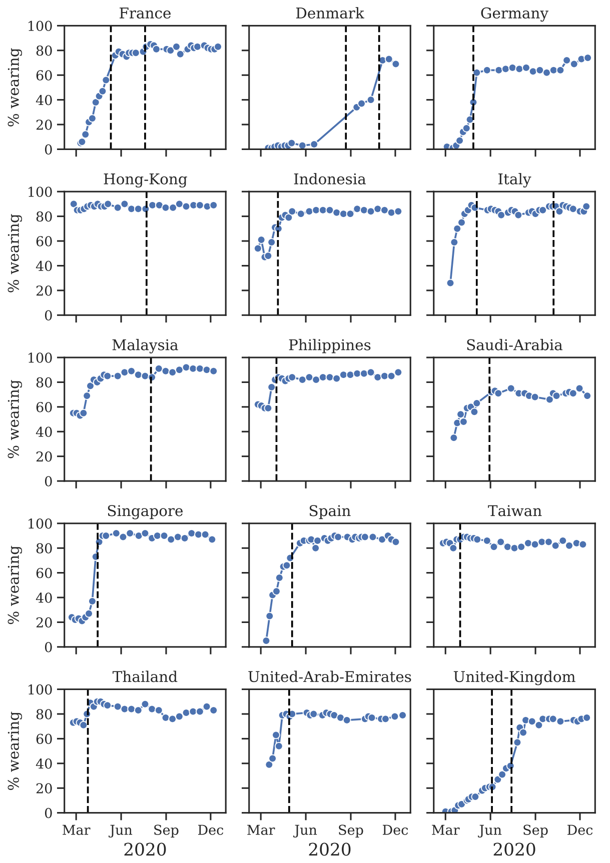

Fig. S.2. YouGov wearing estimates over time, with mandates as dashed lines (6). These regions are those with both YouGov estimates and national mask mandates.

infection-fatality ratio).

The random walk on R captures changes in ascertainment: such AR changes show up as a constant change in cases, and these constants cannot affect the wearing effect, because a change in R from changes in wearing have exponential consequences for cases - see the *Infection Model* section of the main manuscript. (Consider a situation with ascertainment AR_1 and a true number of active infections N. Confirmed cases are then $AR_1 \times N$. If the rate was instead AR_2, confirmed cases would rise by only $(AR_2 - AR_1)N$.)

Further, in most regions, there is often a delay of weeks between a new infection and an update in the confirmed case count. We handle this with the case confirmation delay distribution (see Methods).

We also simulate changing ascertainment rates and thus test this directly. An ascertainment rate below 1 (which is always the case in real data) means that confirmed cases undercount true cases. In this simulation we thus want to scale confirmed cases by a factor above 1. We want the simulated AR to sometimes increase (as regions react to the pandemic and create systems, e.g. testing all hospital admissions) and sometimes decrease (as these systems suffer faults or test shortages, or as behaviour fatigue leads to decreased public subscription to contact tracing apps or test requirements). We choose a relatively long timescale of weeks for these to change.

We simulate changing ascertainment by scaling confirmed cases by the following time-varying process, which adds slowly-varying Gaussian noise $l(t)$ with timescale τ and marginal variance σ^2:

$$\mu_{t+1} = \left(1 - \frac{1}{\tau}\right) l(t) \qquad [1]$$

$$l(t+1) = \mathcal{N}\left(\mu_{t+1}, \frac{2\sigma^2}{\tau}\right) \qquad [2]$$

Letting $l(0) = 0$. We then perturb the confirmed cases by taking $y'_t = y_t \times \exp(1 + l(t))$.

We choose the variance as to ensure the scaling factor remains larger than 1: the spread of ascertainments that result from this process are shown in Figure S.3.

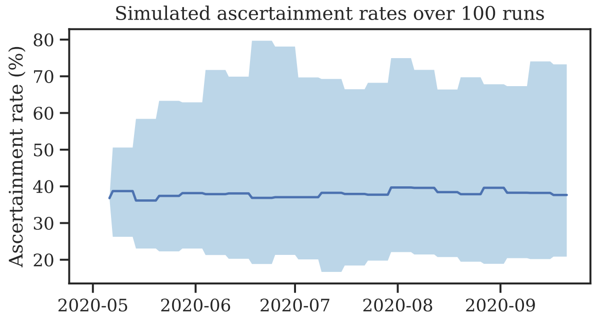

Fig. S.3. Distribution of ascertainment rates implied by our noise process on cases. The flat line is the median of many runs; it is unlikely that any single realisation would resemble it.

The sensitivity analysis in Figure S.38 shows that the estimates are robust to changing ascertainment, though the high variance condition does widen the posterior.

Immunity. One approach to understanding immunity is to estimate the total number of people infected to date, by estimating the case ascertainment rate. However, estimating case ascertainment is difficult. Alternatively, we could use antibody testing data of a random sample of the population. This is not available for most countries during our window of analysis. What data is available indeed suggests very low levels of immunity. For instance, the UK Office for National Statistics COVID infection survey data (based on antibody testing) begins in December 2020 (after the end of our study period, in September 2020) (14). In *December* 2020, 7.5% of people tested positive for antibodies in England. This is already small, and we would expect an even smaller number to have been infected and obtained immunity by September 2020 (the end of our study window). Many of those will have obtained that immunity after our study period, as there was a large surge in cases during that period.

Belgium, one of the few other countries to report mid-2020 antibody testing, reported 6% antibody seropositivity at the end of June 2020 (15). This constitutes something like an upper bound on the international immunity rate, since Belgium was one of the hardest-hit countries in the first wave.

For a confounding influence on our estimates, what matters is not the total amount of immunity, but the *change* in immunity during our study window – and the latter will be even smaller than the numbers stated above.

Thus, changes in R arising from infections are likely to be small, and such changes can be accounted for, at least

in part, by the random walk on R, designed to account for confounding.

The window of analysis used contains negligible numbers of COVID vaccinations. Our results are likely to remain relevant in the mass vaccination period, however: vaccines have not eliminated SARS-CoV-2, and nor is there any evidence that the transmission mechanism has changed dramatically.

B. Mandate data fail to detect the transmission effect

Our main analysis shows that mask-wearing in community settings notably reduces SARS-CoV-2 transmission. If mandates cause wearing, then mandates can reduce transmission, whether by increasing the quantity of people wearing masks, or the quality of mask-wearing (mask type, mask fit, or in more of the venues where transmission is likely to occur).

There are several ways a method could fail to detect this effect. First, since wearing mask-wearing cannot exceed 100% of the population), the marginal effect of a mandate may be limited in cases where wearing was already saturated at a high level at the time of the mandate, even though a mandate could have had a large effect if voluntary wearing had been lower. Second, since the implementation details of mandates are key to their effect (e.g., venues, enforcement intensity, etc.), summaries of average mandate effects can obscure the possibility that some mandates may be highly effective and others not. Third, mandates could increase the length of time (i.e., in months) that people sustain mask-wearing, regardless of strong initial voluntary uptake, but this would not appear in analyses of instantaneous mandate effects. Finally, analyses at the national level can fail to capture sub-national heterogeneity in mandate timing and wearing uptake. In particular, observed weak correlations should not be used to rule out mandate effectiveness in specific contexts. Indeed, natural experiment studies (16, 17) show that mandates can cause wearing in some contexts.

Mask mandates are typically encoded as binary indicators that signal whether mask-wearing was required in at least some shared spaces (18–22). In the main text, we argued that past work using binary mandate data produced inconsistent results because of methodological pitfalls, and so that coarse national mandate data are unsuitable for modelling the wearing-transmission effect. We test this claim by running our model again, using binary mandate data from the Oxford COVID-19 Government Response Tracker (OxCGRT) NPI database (3) instead of UMD self-reported wearing. Only the feature used to represent masks is changed; the priors and functional form are kept the same.

We have two mandate covariates: $x_{\mathrm{ma1},t,c}$ and $x_{\mathrm{ma2},t,c}$. The first covariate, $x_{\mathrm{ma1},t,c}$, represents whether masks were '*required in some or all shared spaces, outside the home with other people present, or some situations when social distancing not possible*' (field H6 from OxCGRT, level 2 (3)). The second covariate, $x_{\mathrm{ma2},t,c}$, has the same conditions, but masking is required in all shared spaces (field H6 from OxCGRT, level 3 or higher). For each mandate type, $x_{\mathrm{ma},t,c} = 1$ if a mask mandate corresponding to the description above is active at time t in region c; otherwise, $x_{\mathrm{ma},t,c} = 0$. $x_{\mathrm{ma1},t,c} = 1$ whenever $x_{\mathrm{ma2},t,c} = 1$, so the correct interpretation of the effect associated with $x_{\mathrm{ma2},t,c}$ is the *additional* effect of mandating masks in all shared spaces, given that mask mandates were already required in some shared spaces.

In this new analysis, we replace $W_{t,c}$ of Infection Model Eq. 2 with

$$\mathrm{Ma}_{\,t,c} = \exp\left(-\alpha_{\mathrm{ma1}}\ x_{\mathrm{ma1},t,c}\right) \cdot \exp\left(-\alpha_{\mathrm{ma2}}\ x_{\mathrm{ma2},t,c}\right)$$

i.e. We model R_t as:

$$R_{t,c} = R_{\mathrm{init},c} \cdot X_{t,c} \cdot \mathrm{Ma}_{\,t,c} \cdot M^{-}_{t,c} \cdot \exp(z_{t,c}).$$

The mandate effect on R is given the prior

$$\alpha_{\mathrm{ma}} \sim \texttt{Normal}(\mu = 0, \sigma = 0.08)$$

Note that the wearing effect prior reflects our beliefs about the effect of going from 0-100% of people likely to wear masks. But in our window, the range of $w_{t,c}$ averages only $\sim 20\%$ across our regions. Accordingly, we choose a mandate effect prior of mandates that has 1/5th of the prior predictive effect as the wearing prior.

All priors remain the same as in the wearing analysis. The resulting mandate effect on transmission is shown in Figure S.4: re-running the same model with mandate data fails to infer an effect.

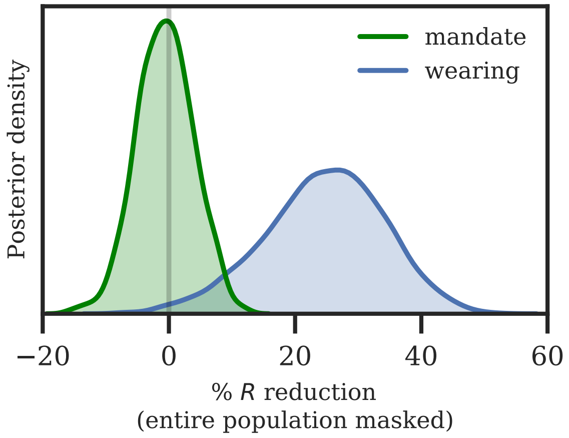

Fig. S.4. Posterior reduction in R using OxCGRT binary mandate data (green) (3). Mandates are operationalised as legally required in at least some public spaces (H6 level 2+) and as required in all public spaces (H6 level 3+). This shows the inadequacy of this data, not the ineffectiveness of mandates.

The above analysis models the effects of mandates on transmission as instantaneous: changing fully on the date the mandate was enforced. This is not an entirely realistic assumption: government policies can have both announcement effects (i.e., people changing their behavior when a new policy is announced but before it is formally implemented) and a gradual adoption (i.e., people gradually changing their behavior in the weeks following implementation), even given mild legal pressure. As a result, we also run the model with a gradual turn-on of the mandate feature, steadily increasing to the maximum value over the week following the beginning of enforcement. This does not notably change the posterior estimate of the mandate effect on R (Fig S.5).

To cover possible announcement effects, we also run the model with a gradual 7-day lead on enforcement, steadily increasing in strength over the week before the beginning of enforcement, until one week after. This also does not notably change the estimated mandate effect (Fig S.6).

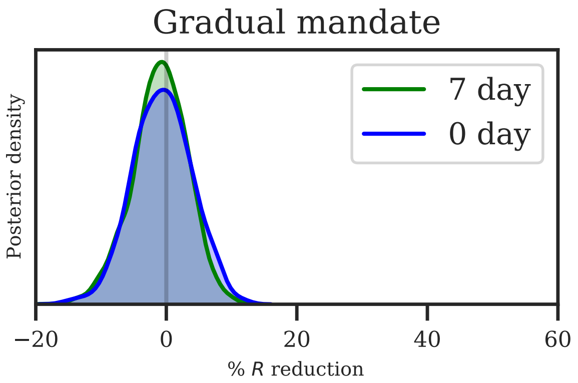

Fig. S.5. Posterior estimate of the mandate effect on R, with the mandate covariate switching on gradually over the first week since enforcement.

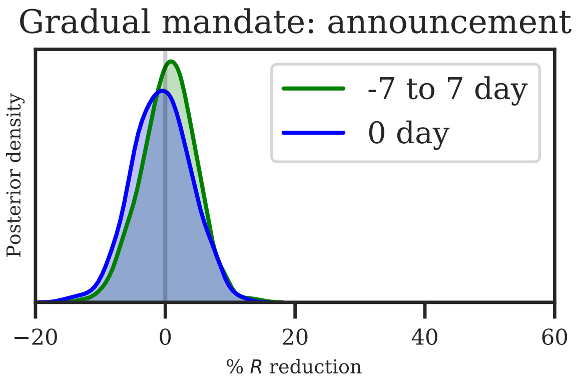

Fig. S.6. Posterior estimate of the announcement mandate effect on R, with the mandate covariate switching on gradually over two weeks, from one week before enforcement to one week after.

Given that wearing substantially reduces transmission, and that mandates increase wearing by 8.6% on average in this window, we might expect to see a small effect of mandates on transmission. Why do we not find this? We hypothesise three reasons. First, the effect we would expect to find is very small–around 2%. Second, the circumstances of mandate policies are highly heterogeneous, both in terms of the preexisting level of voluntary wearing at the time of implementation and in terms of how exactly they are defined, enforced, and complied with. Consequently, averaging the international effect of mandates based on coarse data is unlikely to provide a useful summary of heterogeneous mandate effects. Third, mandate data provide little signal: under half of the regions we study have had mandates, and mandates are one-off; they fail to track day-to-day changes in mask-wearing.

As a result, Fig. S.4 is not a substantive claim about the ineffectiveness of mandates; instead we take it to demonstrate the unsuitability of coarse binary national mandate data for studying mask effectiveness. This has methodological implications: instead of using international data to model the average effects of mandates, researchers should aim to shed light on the conditions under which mandates are likely to be more or less effective at improving and increasing the use of masks.

2020-05-03	Portugal	2020-05-04	Greece
2020-05-04	Nigeria	2020-05-05	New Mexico
2020-05-06	Massachusetts	2020-05-15	Moldova
2020-05-15	Oregon	2020-05-21	Honduras
2020-05-26	South Korea	2020-05-29	Virginia
2020-05-30	Bangladesh	2020-05-30	Saudi Arabia
2020-06-01	Bolivia	2020-06-01	Netherlands
2020-06-02	Panama	2020-06-18	California
2020-06-19	Nebraska	2020-06-22	Costa Rica
2020-06-24	Nevada	2020-06-24	North Carolina
2020-06-24	Utah	2020-06-26	Washington
2020-07-03	Texas	2020-07-06	Switzerland
2020-07-13	Croatia	2020-07-16	Alabama
2020-07-16	Colorado	2020-07-16	Ireland
2020-07-17	Michigan	2020-07-20	Arkansas
2020-07-22	Minnesota	2020-07-22	Philippines
2020-07-27	Indiana	2020-07-28	Paraguay
2020-08-19	Brazil	2020-08-27	Romania
2020-09-01	Czechia	2020-09-01	Italy
2020-09-06	Nepal	2020-09-08	Turkey
2020-09-11	Hungary	2020-09-14	United Kingdom

Table S.4. Enforcement dates for all new national mandates in our window, May-Sep 2020

C. Model Outputs

Visualising the mask effect. To make clearer the magnitude of the mask effect in different regions, we show the estimated effect over time, given local changes in the reported wearing level. All 92 regions are plotted on Zenodo; an example is Figure S.7, chosen to show the effect of a fall in wearing.

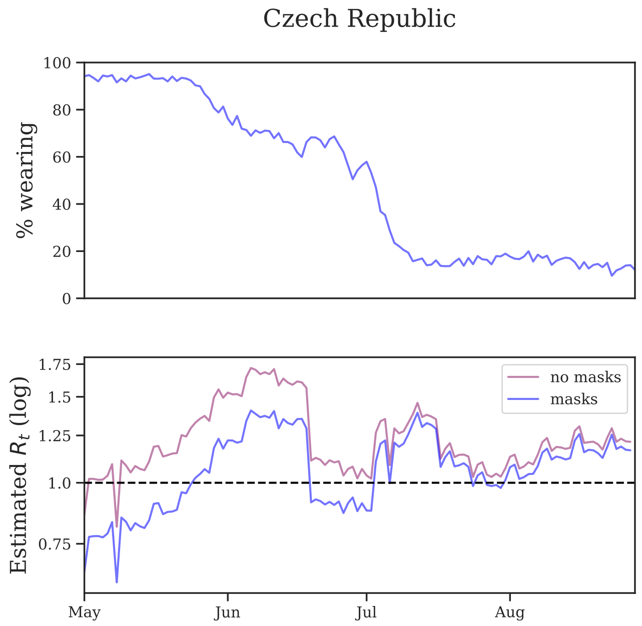

Fig. S.7. Estimated R_t for one of our 92 regions, as the wearing level (top) changed over time. Bottom panel shows the overall R_t estimate (pink) and the estimate excluding the mask effect (blue, simulating a 0% wearing level).

MCMC statistics. We use PyMC3's implementation of Hamiltonian Monte Carlo with the No-U-Turn sampler (NUTS) (23). The following outputs are from running the default model with

the wearing feature.

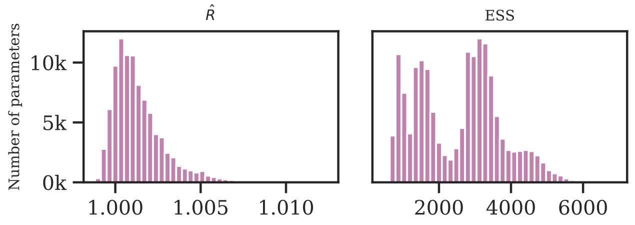

Fig. S.8. Gelman-Rubin $\hat{R}$ score (left) and effective sample size (right) for each parameter in the model.

The Gelman-Rubin diagnostic $\hat{R}$ tests for convergence of the sampler. When $\hat{R}$ is close to 1 (i.e. < 1.01 (24)), the MCMC sampling algorithm is commonly considered to have converged (25). Figure S.8 (left) therefore suggests that our MCMC sampler has converged, and that our posterior may be used to draw valid inferences.

We used 700 tuning samples and 700 posterior samples for each of 4 chains, giving 5600 samples in total. There were no divergent transitions. As shown in Figure S.8 (right), ESS exceeds 30% of the raw sample size for the majority of parameters, indicating low autocorrelation.

Traceplots for the global parameters can be found on Zenodo.

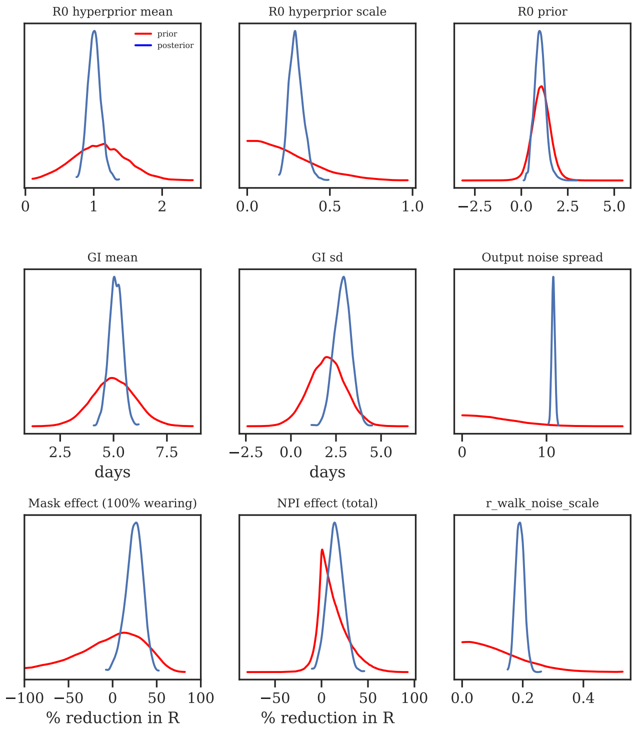

Fig. S.9. Priors vs posteriors for learned model parameters.

Prior-posterior plots. Figure S.9 displays the priors and posteriors for parameters of our model. The posteriors are sharp despite broad priors, which suggests that our data is informative about the parameters.

Prior predictive checks. The high dimensionality of our model makes it difficult to tell what assumptions individual priors jointly imply about the data-generating process. To make our choices interpretable, we inspect the prior predictive curves. Figure S.10 shows draws from nine random regions. This prior seems to capture our beliefs about transmission. In particular, we see step changes in R around the introduction or lifting of NPIs, weekly periodic effects due to changes in mobility, and fluctuations in Rt unlinked to NPIs or mobility. The model allows a wide range of epidemic dynamics: repeated epidemics, the possibility of eradication, and a tendency towards sudden changes induced by binary NPI changes.

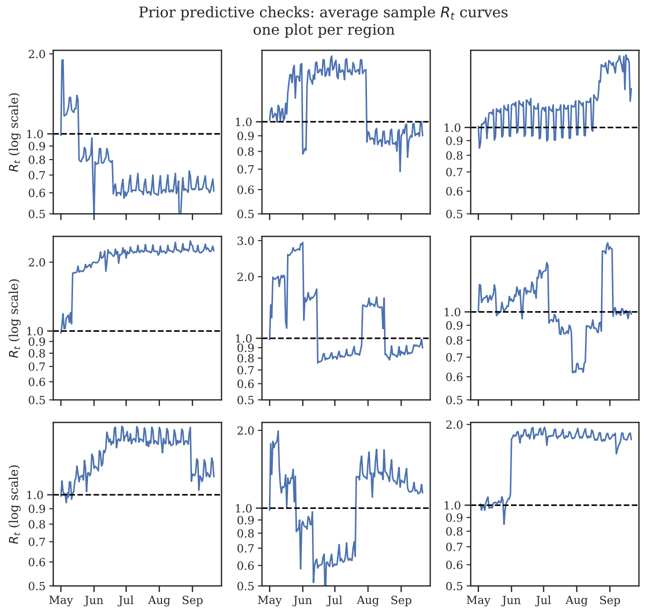

Fig. S.10. Prior predictive checks for a random sample of 9 regions.

Figure S.11 shows the overall prior curve, averaging 1000 draws over all 92 regions (with 95% interval).

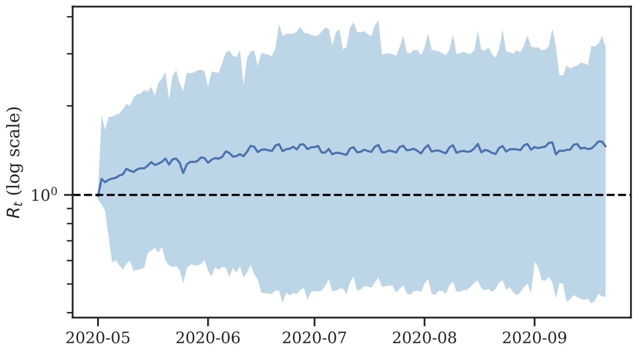

Fig. S.11. Prior predictive check, averaged across regions and 1000 runs.

Posterior predictive distributions. Figure S.12 displays predicted cases during, and 3 weeks beyond, our window of analysis for example regions. All 92 region panels can be found on Zenodo.

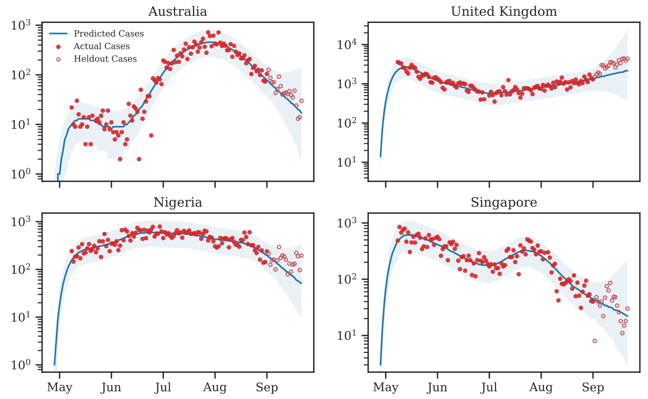

Fig. S.12. Predictive curves from selected regions (log cases on y). The last 20 data points are holdouts, unseen by the model.

Posterior correlations. Figure S.13 shows the posterior correlations between the attributed R reductions for each modelled effect.

We can use these correlations to diagnose excessively strong collinearity in our data; collinearity would manifest as strong posterior correlations (26). However, almost all of the pairwise correlations are $-0.2 < r < 0.1$, which indicates that collinearity is manageable in our dataset. Notable negative effect correlations exist between different levels of the same NPIs:

- Restrictions on gatherings $<$ 100 people and Restrictions on gatherings $<$ 1000 (-0.57);
- Restrictions on gatherings <10 and Restrictions on gatherings <100 (-0.23);
- School reopening (some schools) and School reopening (all schools) (-0.51);

All other pairwise covariate correlations have an absolute value less than 0.2.

Region panels. Figure S.14 displays inferred R_t against covariate values for selected regions with a range of mask-wearing dynamics (low mask wearing and no increase; high wearing and no increase; a gradual increase in wearing; and a sharp increase in mask wearing). All 92 region panels can be found on Zenodo.

D. Sensitivity Analysis

Sensitivity analysis reveals the extent to which results depend on uncertain parameters and modelling choices, and can diagnose model misspecification and excessive collinearity (26). We vary many of the components of our model and recompute the NPI effectiveness estimates. Overall, we perform 20 sensitivity analyses with 60 conditions. Table S.5 summarises our sensitivity analyses and their categories.

The effect sizes inferred for the other NPIs are smaller than in other work (18, 19, 27) because they measure a different effect: in this window, most regions begin with interventions active, and changes in NPI status are most often reopenings/lifting of bans. Such reopenings often result in an increase in transmission that is smaller in magnitude than the decrease

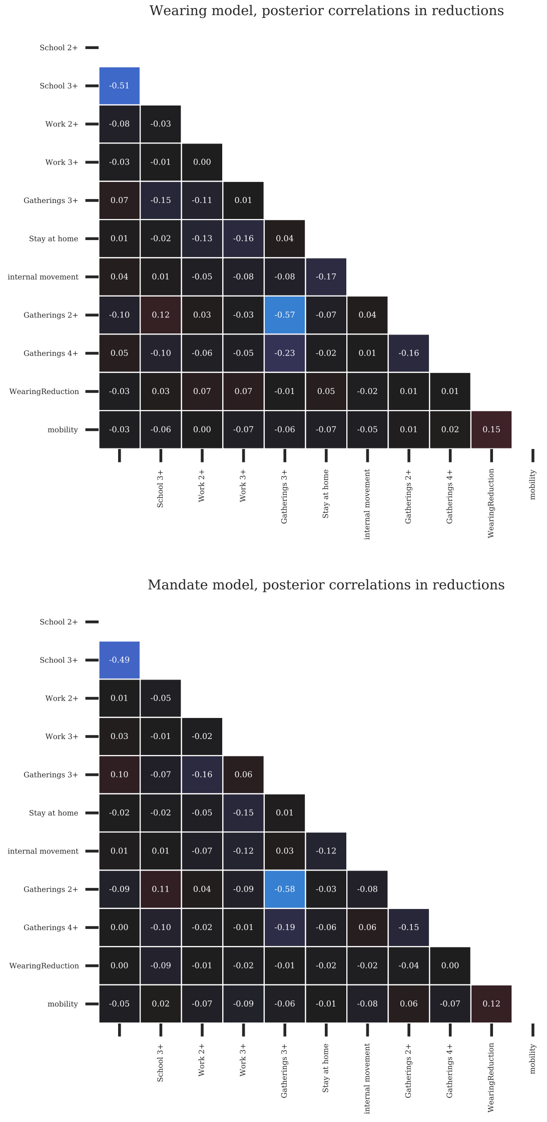

Fig. S.13. Posterior correlations between the covariate effects (reductions in R)

in transmission from the initial policy implementation—for example, due to improved safety procedures (19).

Unobserved factors. Our data do not capture all of the government NPIs that were implemented, and we only measure two forms of voluntary behaviour change: mask wearing and mobility. Unobserved factors may influence R, and if their timing correlates with the timing of mask wearing or mandates, reductions in R from unobserved factors may be wrongly attributed to mask-wearing or mandates (28)—our observed factors will be confounded. For instance, observational estimates like ours are potentially confounded by the correlation between mask-wearing and other protective behaviours (29, 30). We investigate this phenomena by assessing how much effectiveness estimates change when previously observed factors are excluded, following Sharma *et al.* (31).

Figure S.17 shows NPI effectiveness estimates when each observed NPI is excluded in turn. Figure S.18 shows the sensitivity of our effect estimates to excluding mobility from

Category	Experiment type	Description
Unobserved factors	NPI leave-out, Fig. S.17	Each observed NPI is excluded in turn, and then all NPIs are.
	Mobility leave-out, Fig. S.18	Exclude mobility from our model.
	Fake wearing covariate, Fig. S.19	Use a synthetic covariate in place of wearing that captures the wearing trend in each region.
	Mobility and wearing only, Fig. S.20	Exclude all NPIs from the model.
Epidemiological priors	Starting R: mean of hyperprior mean, Fig. S.21	Mean of the prior over the mean of the distribution of country-specific basic reproduction numbers.
	Starting R: scale of hyperprior mean , Fig. S.22	Scale of the prior over the mean of the distribution of country-specific basic reproduction.
	Starting R: scale of prior scale, Fig. S.23	Scale of the prior over the noise on country-specific basic reproduction numbers.
	Random walk noise scale, Fig. S.24	Scale of the prior over the size of the random walk step.
Delay distributions	Generation interval prior mean, Fig. S.25	Mean of the prior over the mean generational interval.
	Confirmation delay mean, Fig. S.26	Mean of the distribution of case confirmation delays.
	Confirmation delay dispersion, Fig. S.27	Dispersion of the distribution of case confirmation delays.
Covariate priors	NPI prior, Fig. S.28	Prior over the NPI effects (not including mask-wearing and mask mandates).
	Wearing effect prior scale, Fig. S.29	Scale of the prior over the wearing effect.
	Mobility effect prior mean, Fig. S.30	Mean of the prior over the mobility effect parameter.
	Mobility effect prior scale, Fig. S.31	Scale of the prior over the mobility effect parameter.
Model structure	Wearing parameterisation, Fig. S.32	The functional form of the mask-wearing effect on R.
	Random walk period, Fig. S.33	Number of days between random walk steps.
Data	Region bootstrap Figs S.34, S.35	Sample 92 regions with replacement from our set.
	Window of analysis, Fig. S.36	Shorter periods of analysis.
	Wearing range, Fig. S.37	Only include regions with $> 15\%$ change in wearing over the window.

Table S.5. Our sensitivity analyses

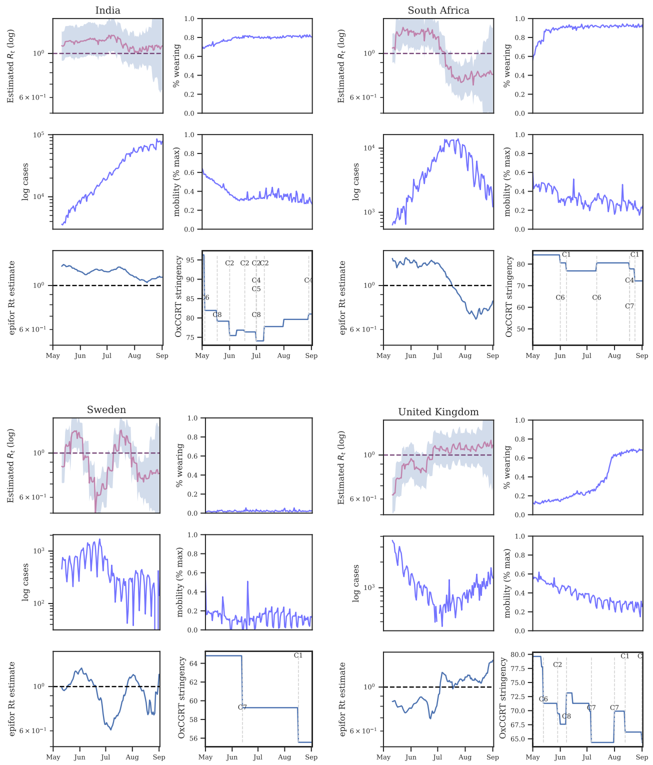

Fig. S.14. Summary plots of selected region covariates and R_t estimates, summer 2020. Top-left: instantaneous R_t from our model. Bottom-left: instantaneous R_t estimates from EpidemicForecasting (12). Bottom-right: overall NPI stringency from OxCGRT (3)

our model. Reducing mobility has a large effect on R, so it is encouraging to see that our effects are robust to excluding mobility from our model.

One objection to our methodology is that mask-wearing increases over our window of analysis while transmission decreases in many regions. It is therefore possible that this correlation is a spurious contributor to the substantial apparent wearing effect. We test this hypothesis by creating a fake wearing variable for each region. Each variable has the same start and end wearing value as the true wearing percentage and linearly interpolates between these values to capture the trend in wearing in that region. We infer a small and uncertain effect for the fake wearing variable 7.6% [–20.2%, 30.0%] (see Figure S.19). This implies that the wearing effect we infer does not rely solely on the wearing trend in this period.

Figure S.20 shows the sensitivity of our effect estimates to excluding all NPIs from our wearing model.

Epidemiological priors. Figure S.21 shows the sensitivity of our effect estimates to $\bar{\mu}$, the mean of the prior over μ in $\tilde{R}_{\text{init},c} \sim N(\mu, \sigma^2)$, where $\mu \sim \text{TruncatedNormal}(0.1, \bar{\mu}, \psi)$. Recall that $\tilde{R}_{\text{init},c}$ is the reproduction number at the start of the window of analysis, supposing mandates are not active and no one is wearing masks. Figure S.22 shows the sensitivity of our effect estimates to ψ the scale of the prior over μ in $\tilde{R}_{\text{init},c} \sim N(\mu, \sigma^2)$, where $\mu \sim \text{TruncatedNormal}(0.1, \bar{\mu}, \psi)$. Figure S.23 shows the sensitivity of our effect estimates to ω, the scale of the prior over σ in $\tilde{R}_{\text{init},c} \sim N(\mu, \sigma^2)$, where $\sigma \sim \text{HalfNormal}(\omega)$. Figure S.24 shows the sensitivity of our effect estimates to the prior over the random walk noise scale.

Delay distributions. Figure S.25 shows the sensitivity of the effect estimates to the mean of the distribution of the genera-

tion interval. Figures S.26 and S.27 show the sensitivity of the effect estimates to the mean and dispersion of the distribution that represents the delay between infection and case reporting.

Covariate priors. Figure S.28 shows the sensitivity of our effect estimates to the prior over the NPI effects. Figure S.29 shows the sensitivity of our effect estimates to the scale of the prior over the wearing effect. Figure S.30 shows the sensitivity of our effect estimates to the mean of the prior over the mobility effect.

Figure S.31 shows the sensitivity of our effect estimates to the scale of the prior over the mobility effect.

Model structure. Figure S.15 shows the effect on R of our three parametrisations of the wearing effect. (The default, reported in all main figures, is the negative exponential, see 'Materials and Methods'.)

Figure S.32 shows the sensitivity of our effect estimates to the parameterisation of the wearing effect. The wearing parameterisations are defined as follows:

- *Exponential* (base model): $W^{\text{exp}}_{t,c} = \exp\left(-\alpha_w w_{t,c}\right)$. We use this form in our base model because it is consistent with the form of the mandate effect on R.
- *Linear*: $W^{\text{L}}_{t,c} = \text{ReLu}\left(1 - \alpha_w w_{t,c}\right)$, where ReLU is the Rectified Linear Unit. The ReLU function preserves positive inputs and maps negative inputs to zero. We include the linear form because it is the simplest way to approximate wearing's effect on transmission.
- *Quadratic*: $W^{\text{Q}}_{t,c} = \text{ReLu}\left(1 - \alpha_{w,1} w_{t,c} - \alpha_{w,2} w^2_{t,c}\right)$. The quadratic form is based on a simple model: suppose two people interact, and there is a fixed, independent probability that each of them wears a mask. Then the reduction in the probability of transmission due to mask-wearing is quadratic in the probability that each wears a mask. The two α parameters correspond to source control and wearer-protection.

Figure S.33 shows the sensitivity of our effect estimates to the period of the random walk. For a period of N days the value of $R_{t,c}$ may change without a change of covariates every N days.

Data permutations. Figures S.34 and S.35 show the sensitivity of our effect estimates to bootstrapping our regions. Bootstrapping assesses how much our effect estimates depend on the regions we included. For each seed we sample 92 regions with replacement from our set of 92 regions. Each bootstrap contains 58/92 *unique* regions on average.

Figure S.36 shows the sensitivity of our effect estimates to shorter periods of analysis. We see little variation in our effect estimates, which implies that our results may generalise to other periods.

Figure S.37 shows the sensitivity of our effect estimates to limiting the sample to regions where wearing varied greatly, i.e. by more than 15% of population over the window of analysis. This drops 26 of the 92 regions, and produces a similar posterior estimate of the wearing effect.

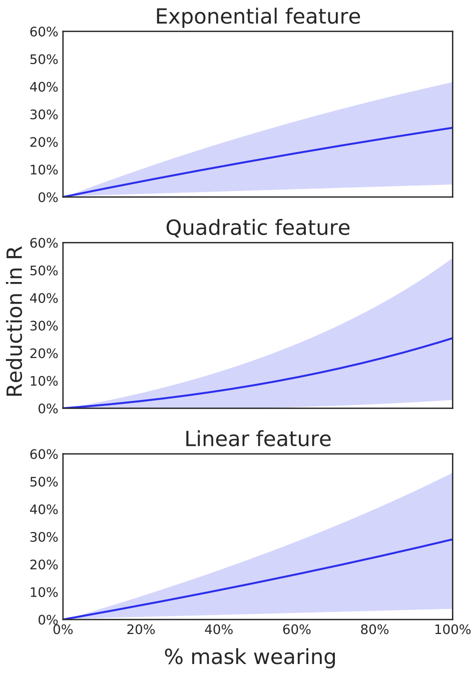

Fig. S.15. Reduction in R over wearing level for our three tested parametrisations, with 95% credible intervals.

1. E Dong, H Du, L Gardner, An interactive web-based dashboard to track COVID-19 in real time. *The Lancet infectious diseases* **20**, 533–534 (2020).
2. H Ritchie, et al., Coronavirus (COVID-19) Vaccinations. https://ourworldindata.org/covid-vaccinations (2021).
3. T Hale, et al., A global panel database of pandemic policies (Oxford COVID-19 government response tracker). *Nat. Hum. Behav.* **5**, 529–538 (2021).
4. F Kreuter, K Stewart, A Garcia, Y Li, J O'Brien, COVID-19 World Symptoms Survey (https://gisumd.github.io/COVID-19-API-Documentation/docs/home.html) (2020).
5. N Barkay, et al., Weights and methodology brief for the COVID-19 Symptom Survey by University of Maryland and Carnegie Mellon university, in partnership with Facebook. *arXiv preprint arXiv:2009.14675* (2020).
6. SP Jones, ICLBDA Unit, Y Plc., Imperial College London YouGov Covid 19 Behaviour Tracker (https://github.com/YouGov-Data/covid-19-tracker) (2020).
7. UK Office for National Statistics, Coronavirus and the social impacts on great britain: Statistical bulletins (2020).
8. DC Farrow, LC Brooks, RJ Tibshirani, R Rosenfeld, Carnegie Mellon University COVIDcast (https://delphi.cmu.edu/covidcast/) (2020).
9. B Rader, et al., Mask-wearing and control of SARS-CoV-2 transmission in the USA: a cross-sectional study. *The Lancet Digit. Heal.* **3**, e148–e157 (2021).
10. Google LLC, Google COVID-19 community mobility reports (https://www.google.com/covid19/mobility/) (2020).
11. HJT Unwin, et al., State-level tracking of COVID-19 in the united states. *Nat. communications* **11** (2020).
12. J Kulveit, the EpidemicForecasting team, Epidemic forecasting: Country Rt estimates (http://epidemicforecasting.org/) (2020).
13. A Cori, NM Ferguson, C Fraser, S Cauchemez, A New Framework and Software to Estimate Time-Varying Reproduction Numbers During Epidemics. *Am. J. Epidemiol.* **178**, 1505–1512 (2013).
14. O for National Statistics, Coronavirus (COVID-19) infection survey, antibody and vaccination data, UK (https://www.ons.gov.uk/peoplepopulationandcommunity/healthandsocialcare/conditionsanddiseases/data (2020).
15. S Herzog, et al., Seroprevalence of igg antibodies against sars coronavirus 2 in belgium – a serial prospective cross-sectional nationwide study of residual samples (march – october 2020). *medRxiv* , (2021).
16. ME Van Dyke, et al., Trends in county-level COVID-19 incidence in counties with and without a mask mandate—Kansas, June 1–August 23, 2020. *Morb. Mortal. Wkly. Rep.* **69**, 1777 (2020).
17. W Lyu, GL Wehby, Community use of face masks and COVID-19: Evidence from a natural experiment of state mandates in the US. *Heal. affairs* **39**, 1419–1425 (2020).
18. JM Brauner, et al., Inferring the effectiveness of government interventions against COVID-19. *Science* **371** (2021).
19. M Sharma, et al., Understanding the effectiveness of government interventions in Europe's second wave of COVID-19. medRxiv 2021.03.25.21254330v1 [Preprint]. 26 March 2021 (2021).
20. C Kenyon, Widespread use of face masks in public may slow the spread of SARS CoV-2: an ecological study. *medRxiv* (2020).
21. X Chen, Z Qiu, Scenario analysis of non-pharmaceutical interventions on global COVID-19 transmissions. *arXiv preprint arXiv:2004.04529* (2020).
22. CT Leffler, et al., Association of country-wide coronavirus mortality with demographics, testing, lockdowns, and public wearing of masks. *The Am. journal tropical medicine hygiene* **103**,

2400–2411 (2020).
23. J Salvatier, TV Wiecki, C Fonnesbeck, Probabilistic programming in Python using PyMC3. *PeerJ Comput. Sci.* **2**, e55 (2016).
24. A Vehtari, A Gelman, D Simpson, B Carpenter, PC Bürkner, Rank-normalization, folding, and localization: An improved $\hat{R}$ for assessing convergence of MCMC. *Bayesian Analysis* (2021).
25. SP Brooks, A Gelman, General methods for monitoring convergence of iterative simulations. *J. computational graphical statistics* **7**, 434–455 (1998).
26. CF Dormann, et al., Collinearity: a review of methods to deal with it and a simulation study evaluating their performance. *Ecography* **36**, 27–46 (2013).
27. S Flaxman, et al., Estimating the effects of non-pharmaceutical interventions on COVID-19 in Europe. *Nature* **584**, 257–261 (2020).
28. A Gelman, J Hill, Causal inference using regression on the treatment variable in *Data Analysis Using Regression and Multilevel/Hierarchical Models.* (Cambridge University Press), pp. 167–198 (2007).
29. DK Chu, et al., Physical distancing, face masks, and eye protection to prevent person-to-person transmission of SARS-CoV-2 and COVID-19: a systematic review and meta-analysis. *The Lancet* **395**, 1973–1987 (2020).
30. C Betsch, et al., Social and behavioral consequences of mask policies during the COVID-19 pandemic. *Proc. Natl. Acad. Sci.* **117**, 21851–21853 (2020).
31. M Sharma, et al., How robust are the estimated effects of nonpharmaceutical interventions against COVID-19? *Adv. Neural Inf. Process. Syst. (NeurIPS 2020)* **33** (2020).

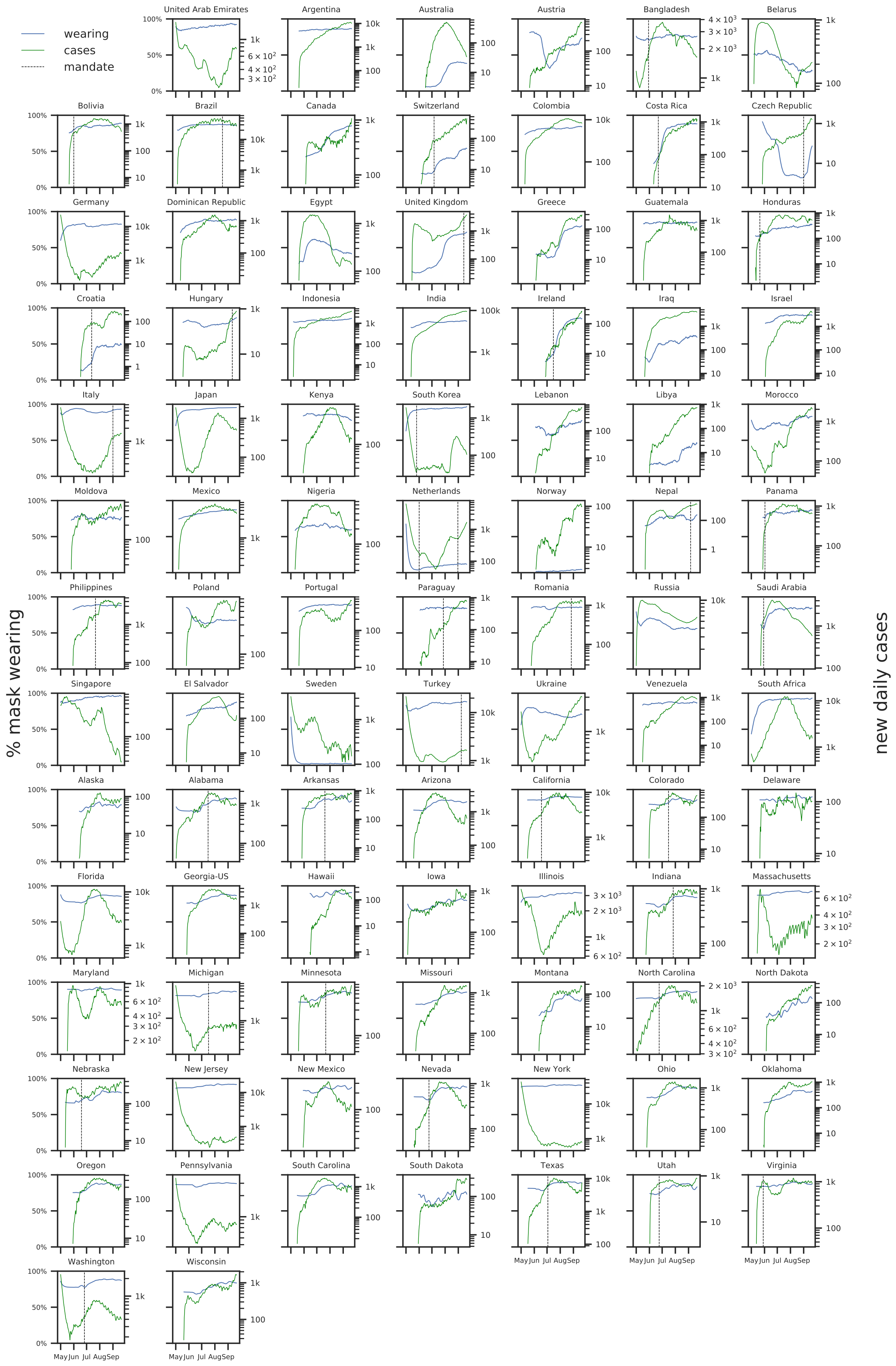

Fig. S.16. Wearing level (blue), log new daily cases (1 week moving average, green), mandate (black) for all regions. For a view including known confounders, see Fig. S.14.

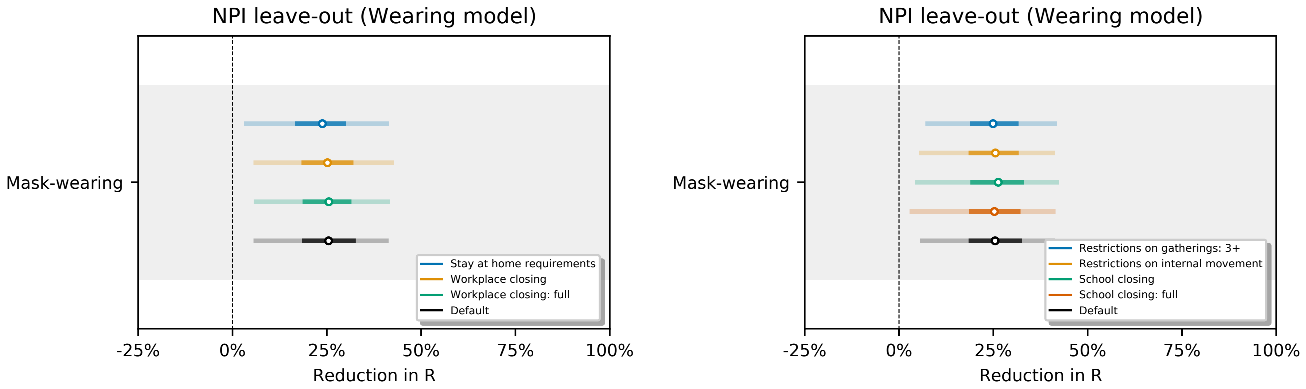

Fig. S.17. Sensitivity of our effect estimates to leaving out recorded interventions, simulating unobserved confounding effects on transmission.

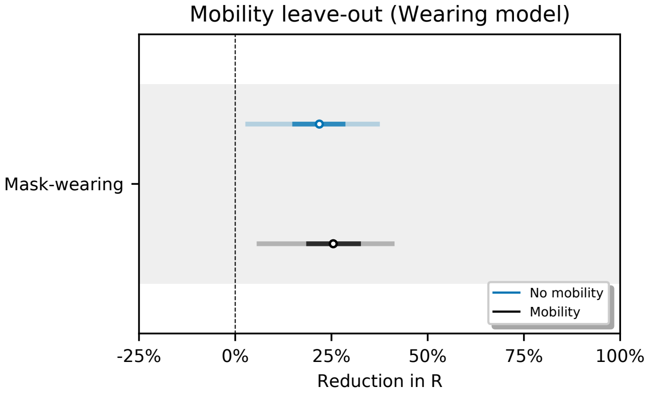

Fig. S.18. Sensitivity of effect estimates to excluding mobility from our model.

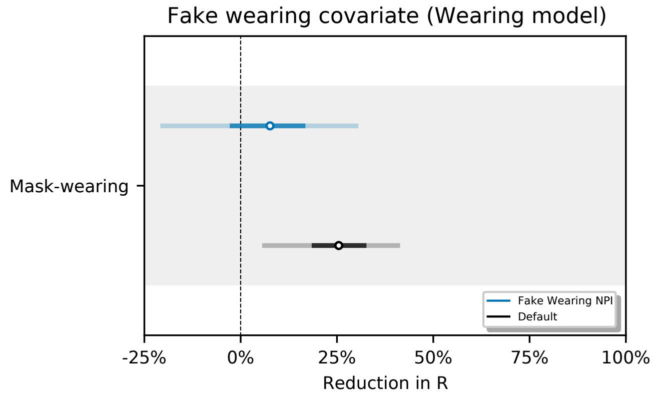

Fig. S.19. Effect estimates when wearing data is replaced by synthetic data that tracks the linear change in wearing, in our window, for each region.

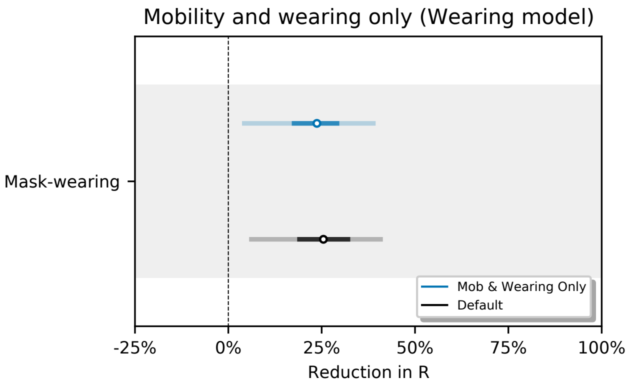

Fig. S.20. Sensitivity of effect estimates to excluding all NPIs.

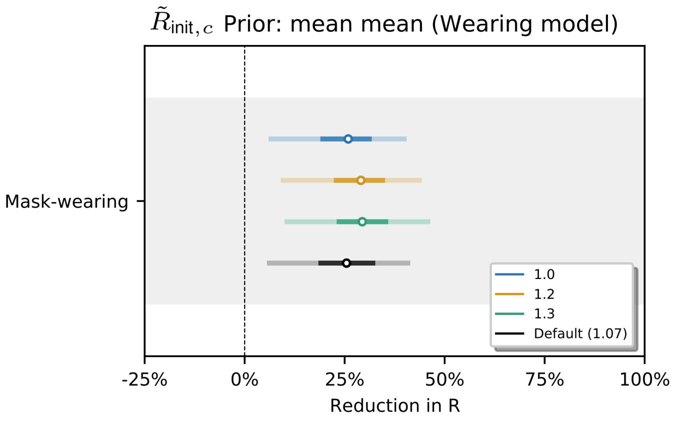

Fig. S.21. Sensitivity of effect estimates to $\bar{\mu}$, the mean of the prior over μ in $\tilde{R}_{\text{init},c} \sim N(\mu, \sigma^2)$, where $\mu \sim \text{TruncatedNormal}(0.1, \bar{\mu}, \psi)$. (L): wearing, (R): mandates.

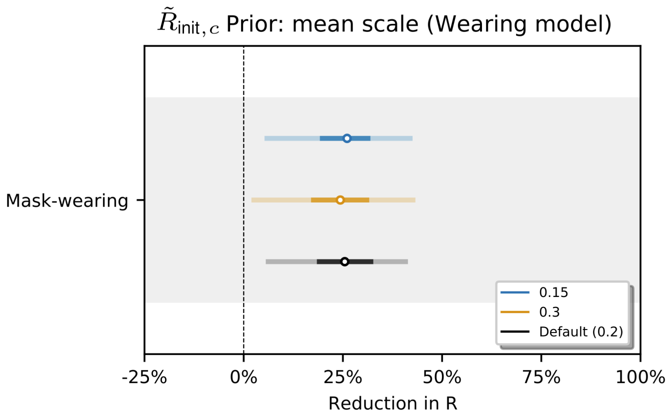

Fig. S.22. Sensitivity of our effect estimates to ψ the scale of the prior over μ in $\tilde{R}_{\text{init},c} \sim N(\mu, \sigma^2)$, where $\mu \sim \text{TruncatedNormal}(0.1, \bar{\mu}, \psi)$.

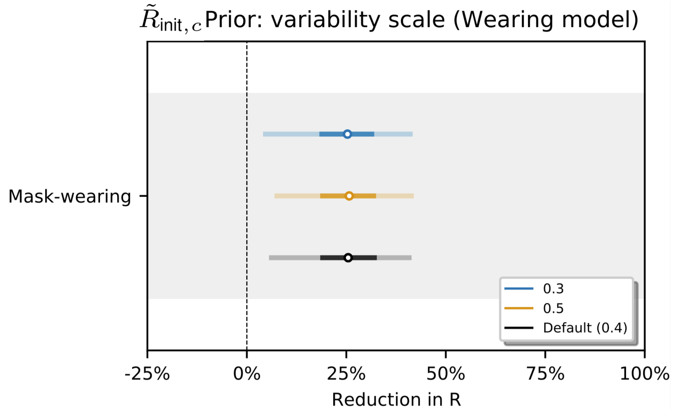

Fig. S.23. Sensitivity of our effect estimates to ω, the scale of the prior over σ in $\tilde{R}_{\text{init},c} \sim N(\mu, \sigma^2)$, where $\sigma \sim \text{HalfNormal}(\omega)$.

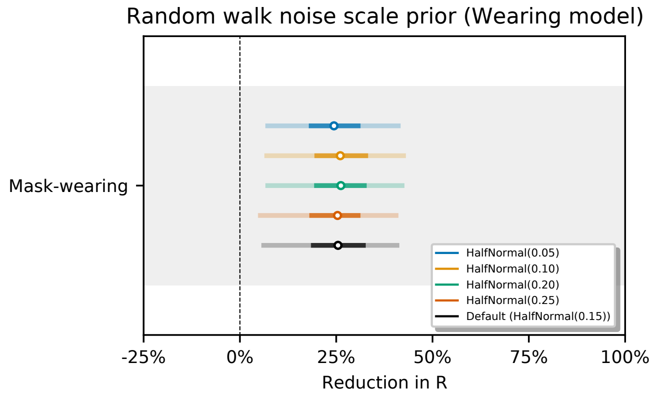

Fig. S.24. Sensitivity of our effect estimates to the noise scale of the weekly random walk.

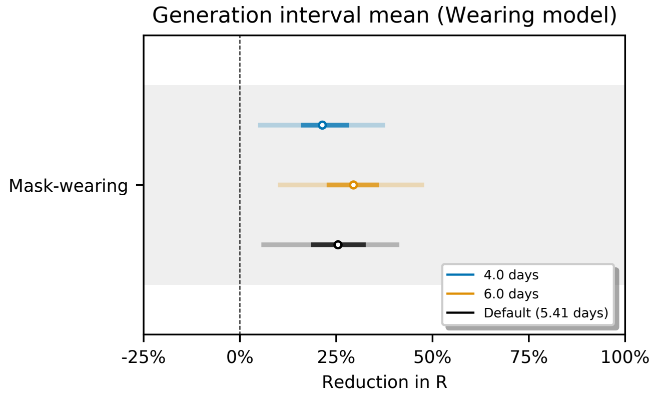

Fig. S.25. Sensitivity of our effect estimates to the mean of the generation interval.

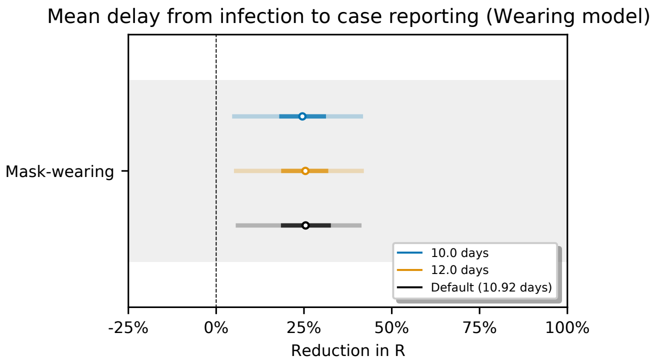

Fig. S.26. Sensitivity of our effect estimates to the mean of the delay from infection to case reporting.

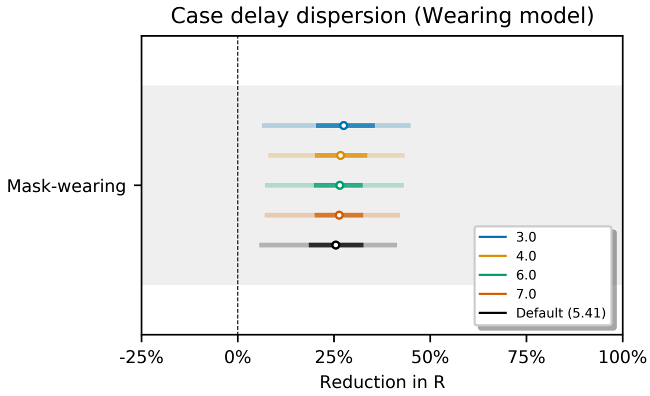

Fig. S.27. Sensitivity of our effect estimates to the dispersion of the delay from infection to case reporting.

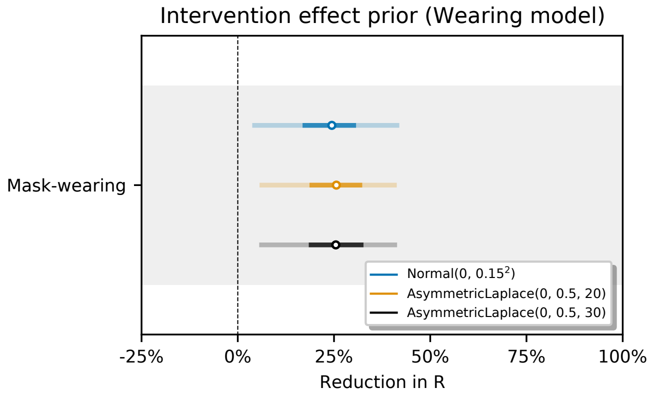

Fig. S.28. Sensitivity of our effect estimates to the prior over the NPI effects.

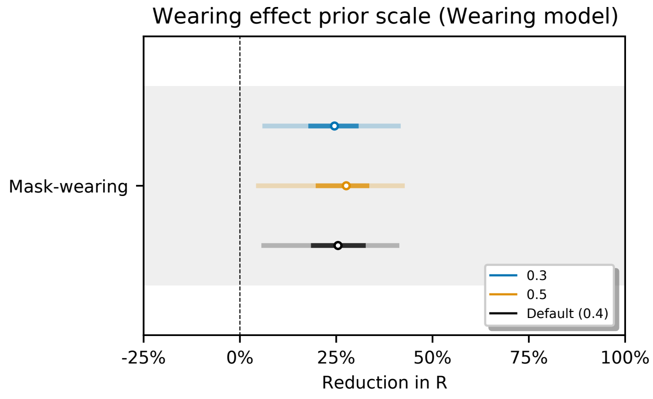

Fig. S.29. Sensitivity of our effect estimates to the scale of the prior over the wearing effect.

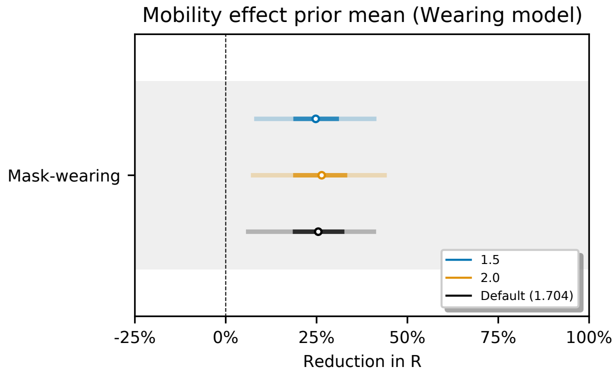

Fig. S.30. Sensitivity of our effect estimates to the mean of the prior over the mobility effect.

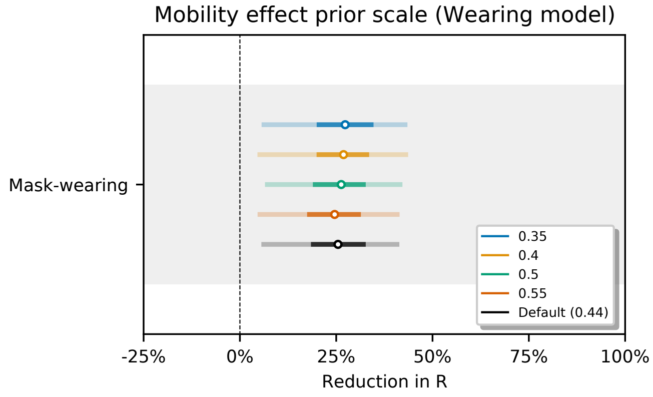

Fig. S.31. Sensitivity of our effect estimates to the scale of the prior over the mobility effect.

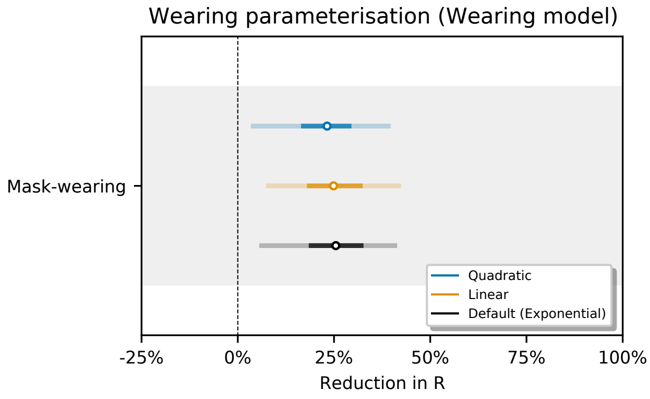

Fig. S.32. Sensitivity of our effect estimates to the to the parameterisation of the wearing effect.

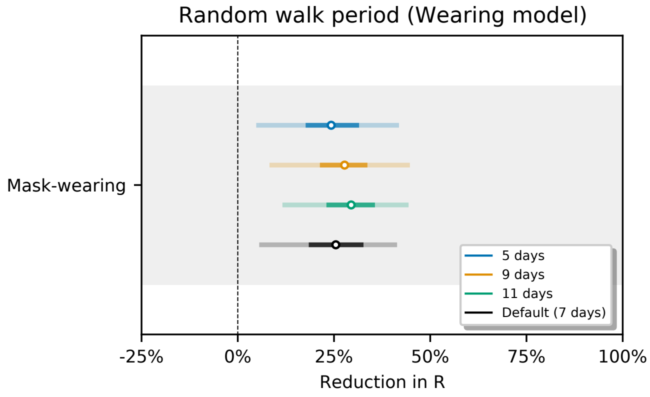

Fig. S.33. Sensitivity of our effect estimates to the period of the random walk.

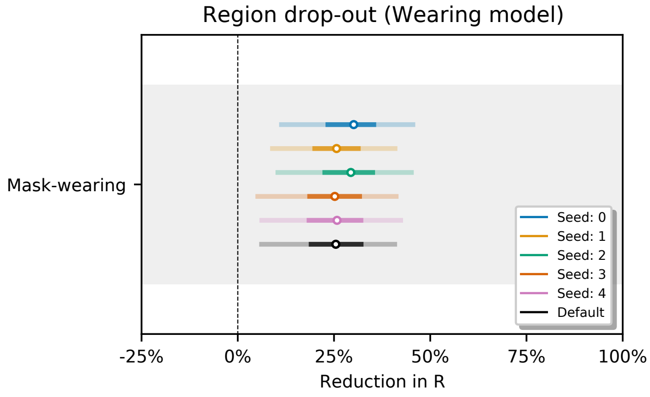

Fig. S.34. Sensitivity of our effect estimates using random bootstrapped sets of regions. Seed 0-4.

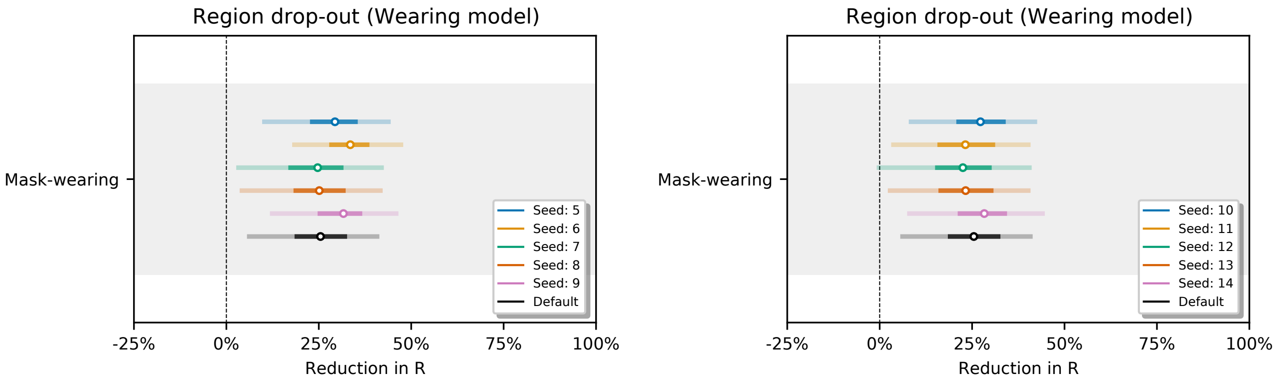

Fig. S.35. Sensitivity of our effect estimates using random bootstrapped sets of regions.

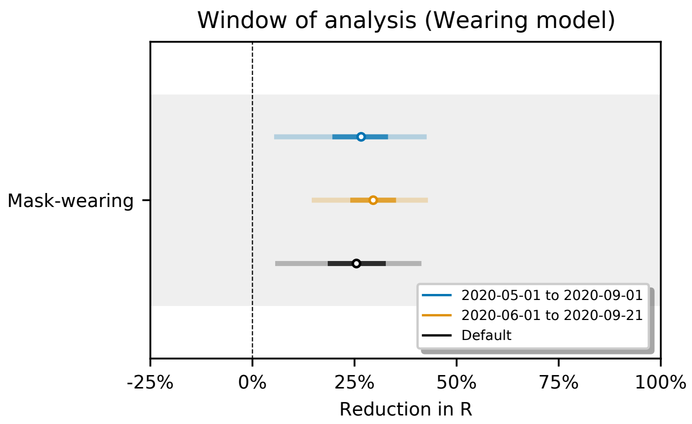

Fig. S.36. Sensitivity of our effect estimates to the window of analysis.

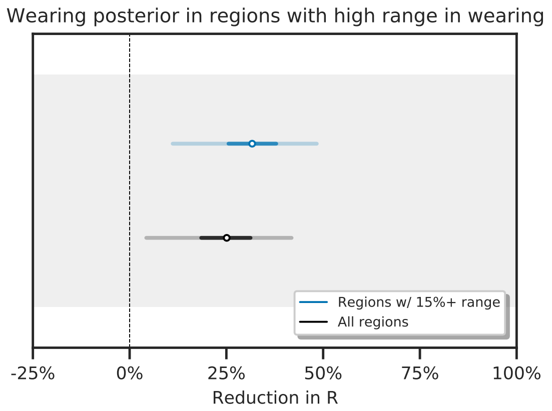

Fig. S.37. Sensitivity of effect estimates to excluding regions where wearing did not vary by more than 15% of population.

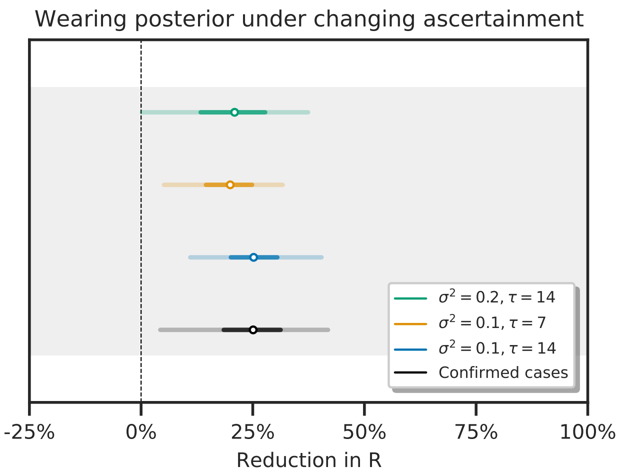

Fig. S.38. Sensitivity of our wearing effect estimate to changes in the rate of unreported cases (time-varying scaling of confirmed cases).
